# Genome-Wide Interaction Analyses between Genetic Variants and Alcohol Consumption and Smoking for Risk of Colorectal Cancer

**DOI:** 10.1371/journal.pgen.1006296

**Published:** 2016-10-10

**Authors:** Jian Gong, Carolyn M. Hutter, Polly A. Newcomb, Cornelia M. Ulrich, Stephanie A. Bien, Peter T. Campbell, John A. Baron, Sonja I. Berndt, Stephane Bezieau, Hermann Brenner, Graham Casey, Andrew T. Chan, Jenny Chang-Claude, Mengmeng Du, David Duggan, Jane C. Figueiredo, Steven Gallinger, Edward L. Giovannucci, Robert W. Haile, Tabitha A. Harrison, Richard B. Hayes, Michael Hoffmeister, John L. Hopper, Thomas J. Hudson, Jihyoun Jeon, Mark A. Jenkins, Jonathan Kocarnik, Sébastien Küry, Loic Le Marchand, Yi Lin, Noralane M. Lindor, Reiko Nishihara, Shuji Ogino, John D. Potter, Anja Rudolph, Robert E. Schoen, Petra Schrotz-King, Daniela Seminara, Martha L. Slattery, Stephen N. Thibodeau, Mark Thornquist, Reka Toth, Robert Wallace, Emily White, Shuo Jiao, Mathieu Lemire, Li Hsu, Ulrike Peters, CCFR and GECCO

**Affiliations:** 1 Public Health Sciences Division, Fred Hutchinson Cancer Research Center, Seattle, Washington, United States of America; 2 Division of Genomic Medicine, National Human Genome Research Institute, Bethesda, Maryland, United States of America; 3 Huntsman Cancer Institute, University of Utah, Salt Lake City, Utah, United States of America; 4 Epidemiology Research Program, American Cancer Society, Atlanta, Georgia, United States of America; 5 Department of Medicine, School of Medicine, University of North Carolina, Chapel Hill, North Carolina, United States of America; 6 Division of Cancer Epidemiology and Genetics, National Cancer Institute, Bethesda, Maryland, United States of America; 7 CHU Nantes, Service de Génétique Médicale, Nantes, France; 8 Division of Clinical Epidemiology and Aging Research, German Cancer Research Center (DKFZ), Heidelberg, Germany; 9 German Cancer Consortium (DKTK), Heidelberg, Germany; 10 Keck School of Medicine, University of Southern California, Los Angeles, California, United States of America; 11 Division of Gastroenterology, Massachusetts General Hospital and Harvard Medical School, Boston, Massachusetts, United States of America; Channing Division of Network Medicine, Brigham and Women's Hospital and Harvard Medical School, Boston, Massachusetts, United States of America; 12 Division of Cancer Epidemiology, German Cancer Research Center, Heidelberg, Germany; 13 Translational Genomics Research Institute, Phoenix, Arizona, United States of America; 14 Department of Surgery, University Health Network Toronto General Hospital, Toronto, Ontario, Canada; 15 Channing Division of Network Medicine, Brigham and Women’s Hospital, Boston Massachusetts, United States of America; 16 Division of Epidemiology, New York University School of Medicine, New York, New York, United States of America; 17 Melbourne School of Population Health, The University of Melbourne, Melbourne, Australia; 18 Ontario Institute for Cancer Research, Toronto, Ontario, Canada; 19 Epidemiology Program, University of Hawaii Cancer Center, Honolulu, Hawaii, United States of America; 20 Department of Health Sciences Research, Mayo Clinic, Scottsdale, Arizona, United States of America; 21 Dana-Farber Cancer Institute, Harvard School of Public Health, Boston, Massachusetts, United States of America; 22 Harvard Medical School, Department of Epidemiology, Harvard School of Public Health, Boston, Massachusetts, United States of America; 23 Centre for Public Health Research, Massey University, Wellington, New Zealand; 24 Department of Medicine and Epidemiology, University of Pittsburgh Medical Center, Pittsburgh, Pennsylvania, United States of America; 25 Division of Preventive Oncology, National Center for Tumor Diseases and German Cancer Research Center, Heidelberg, Germany; 26 Division of Cancer Control and Population Sciences, National Cancer Institute, Bethesda, Maryland, United States of America; 27 Department of Internal Medicine, University of Utah Health Sciences Center, Salt Lake City, Utah, United States of America; 28 Departments of Laboratory Medicine and Pathology and Laboratory Genetics, Mayo Clinic, Rochester, Minnesota, United States of America; 29 Department of Epidemiology, The University of Iowa, Iowa City, Iowa, United States of America; Case Western Reserve University School of Medicine, UNITED STATES

## Abstract

Genome-wide association studies (GWAS) have identified many genetic susceptibility loci for colorectal cancer (CRC). However, variants in these loci explain only a small proportion of familial aggregation, and there are likely additional variants that are associated with CRC susceptibility. Genome-wide studies of gene-environment interactions may identify variants that are not detected in GWAS of marginal gene effects. To study this, we conducted a genome-wide analysis for interaction between genetic variants and alcohol consumption and cigarette smoking using data from the Colon Cancer Family Registry (CCFR) and the Genetics and Epidemiology of Colorectal Cancer Consortium (GECCO). Interactions were tested using logistic regression. We identified interaction between CRC risk and alcohol consumption and variants in the 9q22.32/*HIATL1* (P_interaction_ = 1.76×10^−8^; permuted p-value 3.51x10^-8^) region. Compared to non-/occasional drinking light to moderate alcohol consumption was associated with a lower risk of colorectal cancer among individuals with rs9409565 CT genotype (OR, 0.82 [95% CI, 0.74–0.91]; P = 2.1×10^−4^) and TT genotypes (OR,0.62 [95% CI, 0.51–0.75]; P = 1.3×10^−6^) but not associated among those with the CC genotype (p = 0.059). No genome-wide statistically significant interactions were observed for smoking. If replicated our suggestive finding of a genome-wide significant interaction between genetic variants and alcohol consumption might contribute to understanding colorectal cancer etiology and identifying subpopulations with differential susceptibility to the effect of alcohol on CRC risk.

## Author Summary

Alcohol consumption and smoking are associated with CRC risk. We performed a genome-wide analysis for interaction between genetic variants and alcohol consumption and cigarette smoking to identify potential new genetic regions associated with CRC. About 8,000 CRC cases and 8,800 controls were included in alcohol-related analysis and over 11,000 cases and 11,000 controls were involved in smoking-related analysis. We identified interaction between variants at 9q22.32/*HIATL1* and alcohol consumption in relation to CRC risk (P_interaction_ = 1.76×10^−8^). If replicated our suggested finding of the interaction between genetic variants and alcohol consumption might contribute to understanding colorectal cancer etiology and identifying subpopulations with differential susceptible to the effect of alcohol on CRC risk.

## Introduction

Colorectal cancer (CRC) is the third-most common cancer in men and the second most common cancer in women worldwide [1]. Both environmental and genetic factors are involved in the development of CRC [2–7]. Since 2007, genome-wide association studies (GWAS) have identified about 50 loci associated with CRC risk[8–11]. However, only a small portion of the familial aggregation of CRC is explained by these identified genetic loci, and additional variants associated with CRC susceptibility are more likely to be identified through analyses of interactions between genes and environmental risk factors [12, 13]. Single nucleotide polymorphisms (SNP) that impact only a subgroup of the population or have opposite effects in different subgroups are likely to produce weak main effects that cannot be easily detected by marginal association testing of the SNPs. However, these variants may be identified by testing for interactions between SNP and environmental risk factors (genome-wide interaction analysis) [14, 15]. These findings may provide etiologic insight into CRC and identify potentially susceptible subpopulations [14, 15].

There is compelling evidence from epidemiologic studies that alcohol consumption and cigarette smoking are associated with risk of CRC [16–25]. Both alcohol consumption and cigarette smoking influence disease risk through pathways involving multiple gene products and regulatory elements, providing potential for biological interactions [26–28]. Accordingly, alcohol consumption and smoking are important lifestyle factors to study interactions with genetic variants. In this study, we performed a genome-wide interaction analysis using the large datasets from the Colon Cancer Family Registry (CCFR) and the Genetics and Epidemiology of Colorectal Cancer Consortium (GECCO) [3] to identify SNPs that modify the effects of alcohol and smoking on CRC risk.

## Results

**Fig 1:**
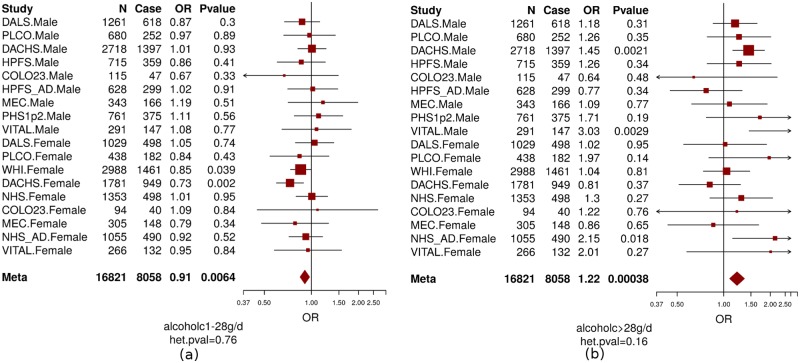
The association between CRC and alcohol consumption (non-/occasional drinkers [reference group]; light-to-moderate drinkers [a]; and heavy drinkers[b]). Men and women were analyzed separately in each study and age and study site (if applicable) were adjusted in model. Non-/occasional drinkers: drinking < 1 gram of alcohol per day; light-to-moderate drinkers: drinking 1–28 grams of alcohol per day ([a] alcoholc1-28g/d); and heavy drinkers: drinking >28 grams of alcohol per day ([b] alcoholc>28g/d). OR: odds ratio; N = total number of subjects; case = number of cases. Colon23: Hawaii Colorectal Cancer Studies 2 and 3; DACHS: Darmkrebs: Chancen der Verhütung durch Screening; DALS: Diet, Activity and Lifestyle Study; HPFS: Health Professionals Follow-up Study; HPFS_AD: Health Professionals Follow-up Study for colorectal adenoma; MEC: Multiethnic Cohort Study; NHS: Nurses’ Health Study; NHS_AD: Nurses’ Health Study for colorectal adenoma; PHS: Physicians’ Health Study; PLCO: Prostate, Lung, Colorectal and Ovarian Cancer; Screening Trial; VITAL: VITamins And Lifestyle; WHI: Women’s Health Initiative. het.pval: p value of heterogeneity.

**Fig 2:**
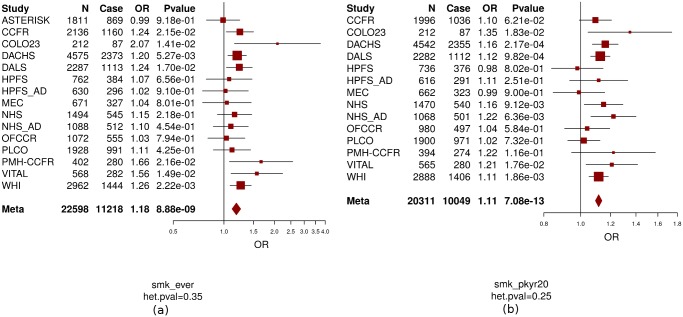
The association between CRC and smoking (ever vs. never smokers [a]; pack-years of smoking [b]). Never smokers were assigned the value 0 for pack-years of smoking. OR: odds ratio; OR for pack-years of smoking is based on per 20 pack-years increase. Age, sex (if applicable), and study site (if applicable) were adjusted in model. ASTERISK: The French Association STudy Evaluating RISK for sporadic colorectal cancer; CCFR: Colon Cancer Family Registry; Colon23: Hawaii Colorectal Cancer Studies 2 and 3.; DACHS: Darmkrebs: Chancen der Verhütung durch Screening; DALS: Diet, Activity and Lifestyle Study; HPFS:Health Professionals Follow-up Study; HPFS_AD: Health Professionals Follow-up Study for colorectal adenoma; MEC: Multiethnic Cohort Study; NHS: Nurses’ Health Study; NHS_AD: Nurses’ Health Study for colorectal adenoma; OFCCR: Ontario Familial Colorectal Cancer Registry; PMH-CCFR: Postmenopausal Hormone study- Colon Cancer Family Registry; PLCO: Prostate, Lung, Colorectal and Ovarian Cancer Screening Trial; VITAL: VITamins And Lifestyle; WHI: Women’s Health Initiative. CCFR is a collaborating study with GECCO. smk_ever: ever smokers; smk_pkyr20: pack-years of smoking; het.pval: p value of heterogeneity.

In this study, we included 14 studies from the Colon Cancer Family Registry (CCFR) and the Genetics and Epidemiology of Colorectal Cancer Consortium (GECCO) as described previously [3, 29, 30] and in the S1 Text and S1 and S2 Tables. Basic characteristics of the participants, stratified by study center, are described in S1 and S2 Tables, respectively. We were able to harmonize measures of alcohol consumption across 8,058 cases and 8,765 controls and measures of smoking across up to 11,219 cases and 11,382 controls. As seen for other common diseases, such as cardiovascular diseases, alcohol consumption shows a different effect with CRC risk depending on the level of alcohol consumed. Heavy alcohol intake (>2 standard drinks per day) has been shown to be associated with increased risk of CRC [16, 17, 31] while light-to-moderate drinking (<2 standard drinks per day) may have little effect [18, 19] or reduce risk of CRC [16, 20–22] compared to non-drinkers. Consistent with these previous publications [16–22, 31] we observed an inverse association with CRC risk for light-to-moderate drinkers (OR = 0.91, P = 0.006, Fig 1A) but a positive association for heavy drinkers (OR = 1.22, P = 0.0004, Fig 1B) compared with non-/occasional drinkers. Modeling alcohol using this categorical approach fitted the association between alcohol intake and CRC risk better than the continuous variable based on the Akaike Information Criterion (AIC) which was 12.42 smaller for the model including the two categorical variables compared with the model including the continuous variable (AIC = 23123.72 for continuous alcohol and AIC = 23111.3 for categorical alcohol)[32]. Given the opposite effect of light/moderate alcohol drinking vs. heavy drinking, it is critical that analyses further investigating the impact of alcohol on CRC, such as interaction analysis do this separately for light/moderate and heavy drinking. Ever-smokers and pack-years of cigarette smoking were positively associated with CRC risk (OR = 1.18 for ever vs. never smokers, P = 8.9×10^−9^; OR = 1.11 per 10 pack-years increase, P = 7.1×10^−13^, Fig 2A and 2B). None of the smoking and alcohol variables showed evidence of heterogeneous associations across studies (P_heterogeneity_>0.16).

**Fig 3:**
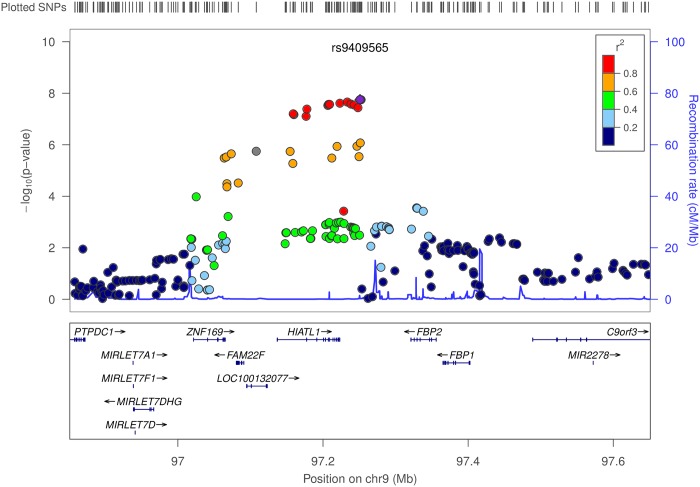
Regional association plot for the interaction analyses between moderate alcohol drinking and SNPs at 9q22.32/*HIATL1*. The–log10 of p values (left y-axis) are plotted against the SNP genomic position based on NCBI build 37 (x-axis); the estimated recombination rate from 1000 Genomes Project European populations are on the right y-axis and plotted in blue. The most significant SNP was denoted with purple diamond. SNPs are colored to reflect correlation with the most significant SNP. Gene annotations are from the UCSC genome browser. Gene *FAM22F* is also known as *NUTM2F*.

**Fig 4:**
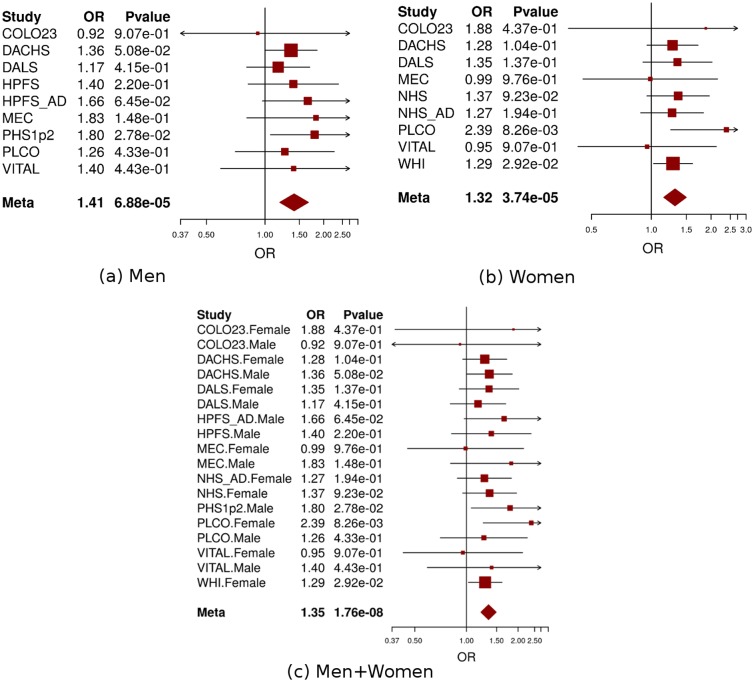
Forest plot for meta-analysis of interaction analysis for rs9409565 and light-to-moderate drinking among men (a), women (b) and combined (c). Odds ratios (ORs) and 95% confidence intervals (95% CI) are presented for the multiplicative interaction between each additional copy of the count (or tested) allele (C) and light-to-moderate vs. non/occasional drinkers. The box sizes are proportional in size to the inverse of the variance for each study, and the lines visually depict the confidence interval. Results from the fixed-effects meta-analysis are shown as diamonds. The width of the diamond represents the confidence interval. P value of heterogeneity for (a), (b), and (c) is 0.93, 0.78, and 0.96, respectively.

Using conventional logistic regression including multiplicative interaction terms, we identified genome-wide significant interactions (at P<5×10^−8^) between 11 SNPs at the 9q22.32/*HIATL1* (Hippocampus Abundant Transcript-Like 1) locus and light-to-moderate drinking with no evidence of heterogeneity across studies (P_heterogeneity_>0.5 for any of the 11 SNPs) (S3 Table, Fig 3). All 11 SNPs were common variants with minor allele frequency (MAF) between 0.31–0.34 and genotyped or imputed with high accuracy (imputation r^2^>0.98, S3 Table). The most significant SNP was rs9409565 with P_interaction_ = 1.76×10^−8^; permuted p-value 3.51x10^-8^ (Table 1, Fig 4C). The genetic variant was located in an intergenic region (28kb downstream of *HIATL1* and 70kb downstream of *FBP2*, Fig 3). All the other 10 genome-wide significant SNPs were in strong linkage disequilibrium (LD) with rs9409565 (LD r^2^>0.8, S3 Table, Fig 3) and some of them were located within the gene *HIATL1*. The observed interaction for rs9409565 was similar in men and women and by cancer site (colon vs rectum) (Fig 4A and 4B, S4 Table). We did not observe any genome-wide significant interaction between any SNP and heavy drinking. No inflation was observed in the genome-wide SNP × alcohol interaction analysis (the inflation factor λ = 0.99 and 1.00 for light-to-moderate drinkers and heavy drinkers, respectively). To evaluate the potential confounding[33] by other lifestyle and environmental risk factors of the interactions between rs9409565 and light-to-moderate alcohol consumption in relation to CRC risk, we adjusted for smoking status (ever vs. never) and BMI (two variables have the highest correlation r = 0.15 and 0.13 with alcohol consumption in our data), as well as exercise, fruit and vegetable consumption in the conventional case-control logistic regression model. Our results did not change (multivariate adjusted interaction p-value = 4.34x10^-8^).

**Table 1:** Stratification analyses^a^ by genotypes of rs9409565 for the association between alcohol consumption and CRC.

Genotype		Non/occasional drinkers			Light-to-moderate drinkers				P _interation_^b^
		Case (n)	Control (n)	OR	Case (n)	Control (n)	OR (95% CI)	P value	
**rs9409565**	CC	1,365	1,593	1.0	1,638	1,717	1.11 (1.00–1.23)	5.9E-02	
	CT	1,495	1,574	1.0	1,646	2,002	0.82 (0.74–0.91)	2.1E-04	
	TT	425	387	1.0	434	590	0.62 (0.51–0.75)	1.3E-06	
									1.76E-08

^a^: non/occasional drinkers as the reference group. Non-/occasional drinkers: drinking < 1 gram of alcohol per day; light-to-moderate drinkers: drinking 1–28 grams of alcohol per day. Men and women were analyzed separately in each study and age, study site (if applicable), and population structure were adjusted in model.

^b^: P value of interaction term between SNP and alcohol consumption, permuted p-value = 3.51x10^-8^ (p value of heterogeneity = 0.96).

When stratified by genotype rs9409565, light-to-moderate alcohol consumption (compared to non/occasional alcohol consumption) significantly decreased CRC risk in individuals with CT genotype (prevalence, 45% vs 49%; OR, 0.82 [95% CI, 0.74–0.91]; P = 2.1×10^−4^) and TT genotype (prevalence, 42% vs 52%; OR,0.62 [95% CI, 0.51–0.75]; P = 1.3×10^−6^) but not in those with CC genotype (P = 0.059) (Table 1, S5 Table). The association between alcohol intake and CRC was also not heterogeneous within each genotype strata (p-heterogeneity > 0.73; S1 Fig).

We also estimated absolute risks of CRC based on Surveillance, Epidemiology, and End Results (SEER) age-adjusted incidence rates (Table 2). Compared with non/occasional drinking, light-to-moderate drinking was associated with 14.0 fewer CRC cases per 100,000 individuals carrying the rs9409565-CT genotype per year; 35.5 fewer CRC cases per 100,000 individuals carrying the rs9409565-TT genotype per year.

**Table 2:** Absolute risk^a^ of CRC for alcohol consumption among individuals with different genotypes of rs9409565.

Alcohol consumption	rs9409565 genotype		
	TT	CT	CC
**Non/occasional drinking**	93.3 (79.9–106.5) ^b^	79.7 (74.0–85.3)	69.7 (64.6–74.8)
**Light-to-moderate drinking**	57.8 (50.1–65.43)	65.7 (61.6–69.9)	77.1 (72.2–82.0)

^a^: Absolute risk calculation was based on Surveillance, Epidemiology, and End Results (SEER) age-adjusted CRC incidence rates between 1982–2011 among the White population of 74.5 per 100,000 men and women per year.

^b^: the number of CRC cases per 100,000 individuals (95% CI).

Using the Cocktail method as a two-step method that may improve power we did not observe any genome-wide significant SNP×alcohol interactions. Further, we did not observe any genome-wide significant interactions for SNP×smoking (smoking history and pack-years of smoking) using logistic regression or the Cocktail method.

### Gene expression analyses

The SNP rs9409565 showing a significant interaction with alcohol is located in an intergenic region between *HIATL1* and *FBP2*. As there is a recombination hotspot lying between rs9409565 and *FPB2* (Fig 3), we focused the gene expression analysis on *HIATL1*, which is expressed in normal colon and rectal tissue. [34, 35] Furthermore, based on our gene expression data for 35 colorectal cancer cases (S2 Text), the expression levels of the *HIATL1* gene was significantly higher in tumor tissues compared with adjacent normal tissues (paired student t test, P<7.2×10^−5^, S2 Fig). This finding is consistent with a previous study [36] which is included in the UCSC Cancer Genomics Browser[37–39] and show that human colon tumors (n = 100) significantly over-expressed *HIATL1* compared to normal colon tissues (n = 5) [36] (Fisher exact test: P = 0.03). Similarly, we were able to reproduce this observation in 50 independent paired colorectal adenocarcinoma and adjacent normal samples from The Cancer Genome Atlas (TCGA) (paired student t test, P = 0.02, S2 Fig). Furthermore, we observed that *HIATL1* showed significant differential expression across various levels of lifetime alcohol consumption in the colon tumor tissues (n = 28, ANOVA test P = 0.03, S3 Fig) and also had differential gene expression across levels of alcohol consumption at reference time (the year before enrollment) in the normal colon tissues (n = 33) at P = 0.06 from ANOVA test (S4 Fig). In addition, for rs9409565 and rs9409567 (LD r^2^ = 1.0 in CEU population), the two most significant SNPs at 9q22.32/*HIATL1*, are cis-acting quantitative trait loci (eQTL) for *HIATL1* expression in lymphoblastoid cell lines (P<7.0×10^−6^) and monocytes (P<5.8×10^−12^) [40, 41], which is consistent with previously published eQTL results from GTEx, Genevar[42], Westra et al., and Lappalainen et al. showing that this these SNPs tag an eQTL locus in lymphoblastoid cells and related anatomical sources (including spleen, whole blood, esophagus muscularis, and sun-exposed skin) with p values ranging from 7x10^-138^ to 4x10^-6^ (S8 Table). In contrast, evaluation of eQTL in both normal (GTEx) and cancer colorectal tissue from TCGA for the rs9409565 locus (r^2^> = 0.2 in Phase 3 1000 genomes EUR data) did not show any significant eQTL. The inability to detect an eQTL is likely because the enhancer tagged by the locus is active in some but not all cancer cell lines and the current reference cancer transcriptome data may not be large enough or molecularly representative of our study population S5 Fig). Furthermore, we investigated whether any of the tagging SNPs are located in variant enhancer loci (VEL)reported by Akhtar-Zaidi et al.[43] using ChIP-seq (H3k27ac) enhancer signals. We observed that four of the variants (rs28406858, rs7042481, rs7858082, and rs9409510) in LD with rs9409565 (LD r^2^≥0.6) were positioned within three gained cancer-specific VEL (S6 Fig).

## Discussion

We identified a suggestive interaction between variants at 9q22.32/*HIATL1* and light-to-moderate alcohol consumption in relation to CRC risk. This is the first genome-wide significant GxE interaction reported for alcohol intake and risk of CRC and warrants replication in independent studies. Evidence for overlap between the discovered 9q22.32/*HIATL1* region with VEL as well as gene expression results support the relevance of the 9q22.32/*HIATL1* region for CRC risk.

Gene expression analyses indicated that a) SNPs identified in our study impact *HIATL1* expression, b) *HIATL1* is involved in signaling pathways related to CRC and expression differs between normal and tumor CR tissue, and c) *HIATL1* expression in colon tissue differs by alcohol consumption. The most significant variant rs9409565 is correlated with 142 variants (LD r^2^≥0.5 in Phase 3 1000 Genomes European populations), which spanned across intronic regions and approximately 50kb downstream and 75kb upstream of *HIATL1*. Nine of these variants (including rs9409550, rs4744345, rs9409546, rs9409778, and rs639276, all with interaction P<5×10^−8^) fall within a transcriptionally active region in normal colon, rectal and duodenal mucosa [44] as defined by epigenetic signals.[45] Furthermore, these variants fall in a region of enriched enhancer signal; although we note that currently available ChIP-seq data are not able to identify a putative transcription factor binding site at any of the tagged SNPs (S6 Fig). In support of our findings that HIATL1 expression is higher in tumor than adjacent normal colorectal tissue, ChIP-seq (H3k27ac) enhancer signals suggest that this locus implicates a gained enhancer present in CR tumors that is absent in normal crypt cells (S6 Fig). In summary, multiple data points suggest that the genetic variants we identified to interact with alcohol on CRC risk are located in regulatory regions impacting the expression of *HIATL1* and that *HIATL1* expression varies by alcohol consumption.

*HIATL1* is a member of the solute carrier (SLC) group of membrane transport, which enables the directed movement of substances (such as peptides, amino acids, proteins, metals, and neurotransmitters) into or out of cells and plays an important role in a variety of cellular functions [46, 47]. Although the detailed function of *HIATL1* remains elusive, this gene was found to be expressed in a large range of animal species and it is highly evolutionarily conserved [48], suggesting an potentially important functional role. Transporter proteins are commonly upregulated in many cancers [49, 50] and take part in nutrient signaling to the mTOR pathway [51] which is an important signaling pathway in apoptosis and cancer [52–54]. Alcohol may modify the effects of *HIATL1* on CRC risk through its influence on the gene expression of *HIATL1*. Nonetheless, the precise mechanism(s) of the interaction between alcohol and *HIATL1* on CRC risk remains unclear and further studies are needed.

Our Cocktail method for detecting G×E interactions did not identify the statistical interaction detected by the conventional logistic regression analysis because rs9409565 did not show strong statistical evidence for association with CRC risk in the marginal association analyses (P = 0.54, OR = 1.014) or with alcohol consumption (P = 0.22). Accordingly, this SNP was ranked low in step 1 of the Cocktail method, resulting in very stringent alpha-threshold for the interaction term in step 2. Although the conventional logistic regression analysis tends to be less powerful overall for genome-wide interaction analysis compared with the Cocktail method [14, 55], it has greater power to detect an association if the marginal association of the SNP on disease or the correlation of the SNP with environmental factor are weak as it was the case for the observed interaction. In addition, no association between rs9409565 and alcohol consumption excluded the possibility that the observed interaction was due to the dependence between them [56]. We also explored the effect of rs9409565 and alcohol using other potentially more powerful single step approaches and observed a similar interaction effect in the Empirical Bayesian analysis[57] and a weaker interaction effect in the case-only analysis[58], which may be explained by the non-significant differential effect of alcohol on CRC in individual carrying the CC genotype (S6 Table).

To investigate if genome-wide interaction may help identifying variants that would be missed we looked up the marginal association of rs9409565 in the largest GWAS[59] which is about twice as large as our study and showed an OR for rs9409565 of 0.975 (95%CI 0.946–1.007, p-value 0.127). Accordingly, the variant by itself showed only weak evidence for association with CRC. This may not be surprising given that it is estimated that the sample sizes required to identify GxE interaction vs. main effects is at least 4x larger[60]. Our study has several strengths, including the large sample size, environmental exposure assessment in well-characterized populations, and standardized harmonization of environmental data across studies. Further, there is no evidence of heterogeneity across studies for our findings, indicating our results are not dominated by one or a few studies and, indeed, represent evidence across all studies. There are also some limitations. Because amassing sufficient study power for genome-wide interaction analysis is a challenge, we combined all studies in the analysis to gain the greatest power[61] instead of dividing studies into discovery and replication sets. Although we do not have a replication set, the consistency of our findings across all studies and the independent evidence from different types of gene expression data and bioinformatics analyses support a novel interaction for CRC risk between alcohol intake and variants in the 9q22.32/*HIATL1* region. Our analyses focused on current alcohol consumption, rather than lifetime alcohol use, which may cause misclassification of a certain portion of alcohol users. Both differential and non-differential misclassifications of alcohol consumption levels tend to lead to underestimation of interaction parameters (e.g. leading to non-significant interaction term between SNP and alcohol intake) [62], accordingly, we may have missed some true interactions. However, it is unlikely that this led to false positives for the interactions observed. Because, there is no strong evidence that the type of alcohol (usually defined as wine, beer and hard liquor) has a differential impact on CRC[63] we have not investigated interaction between genetic variants and type of alcohol. As we preformed genome-wide interaction testing for two environmental risk factors (smoking and alcohol consumption), additional adjustment for multiple comparisons may be needed. However, we note that the observed interaction at 9q22.32/*HIATL1* would remain borderline significant (alpha threshold = 5×10^−8^/2 = 2.5×10^−8^). The small numbers of heavy drinkers, particular in women, impeded the reliable estimation of interaction parameters and limited our power to identify significant interaction between SNP and heavy drinking. We focused gene expression analysis on *HIATL1* because rs9409565 is located in an intergenic region between *HIATL1* and *FBP2* and further there is a recombination hotspot lying between rs9409565 and FPB2. If we expand gene expression analyses for all genes 500kb upstream or downstream 500kb of rs9409565 in the 35 pairs of colorectal tumor-normal tissue samples (S2 Text) we observed no significant result after false discovery rate (FDR) correction. The most significant results were for *MIRLET7F* which has a p value of 0.001 for testing differential gene expression across various levels of lifetime alcohol consumption in normal tissues and *PTPDC1* which has a p value of 0.002 for testing differential gene expression across various levels of alcohol consumption at reference time. Further studies are needed to confirm our findings.

Alcohol has a particularly detrimental effect on several cancers, possibly including CRC, in Asian subpopulations with genetic determined alcohol sensitivity[64–66]. However, as we have focused our analysis on European descent populations and did not observe significant differences of the alcohol-CRC association between studies (phet = 0.16–0.76) we do not expect major underlying differences of the effect of alcohol in our study populations.

We did not perform stratification analyses by anatomical sites for our genome-wide GxE interaction analysis because the association of CRC with alcohol consumption (S7 Table) and smoking [23] did not vary according to anatomical site within the large bowel. Although we did observe potential interactions for alcohol consumption, we did not observe statistical evidence for genome-wide SNP x smoking interactions. This may be because smoking has a weaker association with CRC compared with alcohol intake [24, 26, 67], so we may have been underpowered even with more than 10,000 cases and 10,000 controls. We also may not have properly captured the most relevant smoking variables, such as duration of smoking or time since quitting smoking. The association between smoking and CRC risk are strongest for tumors that display certain molecular features such as microsatellite instability (MSI)-high and CpG island methylator phenotype (CIMP)-positive [68, 69]. Because of the lack of MSI or CIMP data in several studies, we cannot perform stratification analysis by tumor characteristics for smoking-related analyses.

We note that it would be too early to make any recommendation on alcohol intake from our findings even after independent replication given that such recommendation need to be considered in context of the effect of alcohol on all diseases. Furthermore, it will be important to investigate the interactions between alcohol and genetic variants in larger studies to comprehensively evaluate the full impact of genetic variation on the effect of alcohol on colorectal cancer risk.

In summary, we identified a tentative novel interaction for CRC risk between alcohol intake and variants at 9q22.32/*HIATL1*. Further replication and functional studies are required to confirm our findings and understand the biologic implications of the interaction. This, in turn, could provide further insight into CRC etiology and may identify potentially susceptible subpopulations.

## Materials and Methods

### Ethics statement

The overall project was reviewed and approved by the Fred Hutchinson Cancer Research Center Institutional Review Board (approval number: 6501 and 3995). Each study was approved by the local IRB [University of Hawaii Human Studies Program (Colo23 and MEC); University of Utah Institutional Review Board (DALS); Partners Human Research Committee (NHS and PHS); Harvard School of Public Health Institutional Review Board (HPFS); Fred Hutchinson Cancer Research Center Institutional Review Board (VITAL, overall study); Ethics Committee of the Medical Faculty of the University of Heidelberg (DKFZ); NCI Special Studies Institutional Review Board (PLCO)]. For each participating study, participants or the next of kin in the case of deceased participants, provided either written informed consent to participate (Colo23, DACHS, DALS, MEC, PHS, PLCO, VITAL, WHI) or they provided implied written consent by the return of the mailed questionnaires (NHS, HPFS). Additional consent to review medical records was obtained through signed written consent.

### Study population

We included 14 study centers from the CCFR and GECCO as described in the S1 Text and S1 and S2 Tables. All colorectal cancer cases were defined as colorectal adenocarcinoma and confirmed by medical records, pathologic reports, or death certificates. We included advanced colorectal adenoma, a well-defined colorectal cancer precursor [70, 71], from two studies (S1 Text). Advanced adenoma was defined as an adenoma 1 cm or larger in diameter and/or with tubulovillous, villous, or high-grade dysplasia/carcinoma-in-situ histology. Colorectal adenoma cases were confirmed by medical records, histopathology, or pathologic reports. Controls for adenoma cases had a clean sigmoidoscopic or colonoscopic examination. All participants provided informed consent and studies were approved by their respective Institutional Review Boards.

### Genotyping, quality assurance/quality control and imputation

Average sample and SNP call rates, and concordance rates for blinded duplicates have been previously published [3]. In brief, genotyped SNPs were excluded based on call rate (< 98%), lack of Hardy-Weinberg Equilibrium in controls (HWE, p < 1 x 10^−4^), and low minor allele frequency (MAF<0.05). We imputed the autosomal SNPs of all studies to the Northern Europeans from Utah (CEU population) in HapMap II. SNPs were restricted based on per-study minor allele count > 5 and imputation accuracy (R^2^ > 0.3). After imputation and quality-control (QC) exclusion, approximately 2.7M SNPs were used in analysis.

All analyses were restricted to individuals of European ancestry, defined as samples clustering with the Utah residents with Northern and Western European ancestry from the CEPH collection population in principal component analysis [72], including the HapMap II populations as reference.

#### Alcohol consumption and smoking information

All information on basic demographics and environmental risk factors were collected through interviews or through self-administered questionnaires. Data for all studies were centrally harmonized at the data coordinating center. We used the risk-factor information at the reference time, which varied across studies (S1 Text). A multi-step data-harmonization procedure which is described in detail in Hutter et al. [29] was applied to reconcile differences in individual study questionnaires. We converted consumption of alcoholic beverages into grams of alcohol per day (g/day) by summing the alcohol content of each beverage consumed per day. To test if the categorical or continuous variable fitted the association between alcohol intake and CRC risk better we used Akaike Information Criterion (AIC) to compare both models. With our sample size a model with an AIC that is 6 points smaller than the other model is considered a better fitting model[32]. According to this analysis and consistent with previously described risk profiles [16, 17, 19–22, 73], we grouped study participants as non-/occasional drinkers (drinking < 1 g/day); light-to-moderate drinkers (drinking 1–28 g/day); and heavy drinkers (drinking >28 g/day, one standard drinking is approximately equal to 14 grams of alcohol). We coded these categories using indicator variables for the genome-wide interaction analysis. Smoking history was defined as never- and ever-smoking; pack-years of smoking was calculated by multiplying the average number of packs of cigarettes smoked per day by smoking duration (years). Smoking history (ever vs. never smoking) and pack-years (treated as a continuous variable) of smoking were used in genome-wide interaction analysis, separately.

### Statistical analysis

Statistical analyses of all data were conducted centrally at the GECCO coordinating center on individual-level data to ensure a consistent analytical approach. Unless otherwise indicated, we adjusted for age at the reference time, sex (when appropriate), center (when appropriate), and the first three principal components from EIGENSTRAT to account for potential population substructure. The alcohol and smoking variables were coded as described above. Each directly genotyped SNP was coded as 0, 1, or 2 copies of the variant allele. For imputed SNPs, we used the expected number of copies of the variant allele (the “dosage”), which has been shown to give unbiased test statistics [74]. Genotypes were treated as continuous variables (i.e. log-additive effects). Each study was analyzed separately using logistic regression models and study-specific results were combined using fixed-effects meta-analysis methods to obtain summary odds ratios (ORs) and 95% confidence intervals (CIs) across studies. We calculated the heterogeneity p-values using Woolf’s test [75]. Quantile-quantile (Q-Q) plots were assessed to determine whether the distribution of the p-values was consistent with the null distribution (except for the extreme tail). Subjects with missing data for SNPs or environmental factors were excluded from the relevant analyses. Considering the potential male-female difference in alcohol metabolism[76, 77] and the different levels of alcohol consumption between sexes, we conducted the genome-wide interaction analysis for alcohol separately for men and women and used fixed effects meta-analysis to combine their results. All analyses were conducted using the R software (Version 3.0.1).

Two statistical methods that leverage SNPs and environmental factors interaction (G×E interaction) were used to detect potential disease associated loci. First, we used conventional case-control logistic regression analysis including G×E interaction term(s). As the alcohol consumption variable has three categories there are two interaction terms in the statistical models. Based on an increasing number of publications [78–83] providing a detailed discussion on the appropriate genome-wide significance threshold, which all arrive at similar values in the range of 5 x 10^-7^to 5 x 10^−8^ for European populations, we decided to use an alpha level of 5 x 10^−8^ as the genome-wide significance threshold, assuming about 1 million independent tests across the genome (0.05/1,000,000 = 5 x 10^−8^). For significant results we used permutation approach to determine the empirical p-value. We defined the number of permutation needed as 1/p-value (i.e., for a p-value of 5 x 10^−8^ 1/5E-08 = 20,000,000). We permutated the case-control status 1/p-value times and calculated the p values for the interaction from each meta-analyses to calculate the permuted p-value.

Second, we used our recently developed Cocktail method.[55] In brief, this method consists of two-steps: a screening step to prioritize SNPs and a testing step for GxE interaction. For the screening step, we ranked and prioritized variants through a genome-wide screen of each of the 2.7M SNPs (referred to as “G”) by the maximum of the two test statistics from marginal association testing of Gs on disease risk [84], and correlation testing between G and exposure (E) in cases and controls combined.[85] Based on the ranks of these SNPs from screening, we used a weighted hypothesis framework to partition SNPs into ordered groups and assigned each group an alpha-level cut-off, with higher ranked groups from the screening stage having less stringent alpha-level cut-offs for interaction [86, 87]. The second step of the Cocktail method is the testing step. We used either case-control (CC) or case-only (CO) logistic regression to calculate a p-value for the interaction. If the G was assigned based on its low marginal association P value in the screening tests, we used CO test; if it was ranked because of a low correlation screening p-value, we used CC tests. We compared the test step p-value to the alpha-level cutoff for each SNP in a given group.

We calculated absolute risks for each genotype of the SNP showing significant G×E interaction. Briefly, based upon the Surveillance, Epidemiology, and End Results (SEER) age-adjusted colorectal cancer incidence rate (denoted by “I”) between 1982–2011 among the White population of 42.9 per 100,000 men and women per year, we estimated the reference incidence rate of colorectal cancer (denoted by “I_{reference}”) using the following formula: I_{reference} = I/(P(AA, non-E) + OR{Aa, non-E}×P(Aa, non-E) + OR{aa, non-E}×P(aa, non-E) + OR{AA, E}×P(AA, E) + OR{Aa, E}×P(Aa, E)) + OR{aa, E}×P(aa, E)), where P(genotype, E (or non-E)) is the prevalence of light-to-moderate drinking (or non/occasional drinking) in each corresponding genotype category among controls (non-cases). Based on this reference incidence rate of colorectal cancer (i.e., I_{reference}), we further calculated absolute colorectal cancer incidence rates within each subgroup defined by genotype of the SNP according to a light-to-moderate drinking or non/occasional drinking by multiplying the I_{reference} with each corresponding OR. Bootstrap methods were used to calculate the 95% CI of absolute risk estimates [88].

### Expression analyses

We used different types of gene expression data to examine putative expression of genes identified in our genome-wide interaction analysis, and to determine biological plausibility that the variants identified might impact CRC risk. First, we searched the Genotype-Tissue Expression project (GTEx) portal (http://www.broadinstitute.org/gtex/searchGenes)[34] and the Human Protein Atlas (http://www.proteinatlas.org)[35] to establish whether the implicated genes and corresponding proteins are expressed in human colon/rectal tissues. Second, we used several eQTL databases including the Browser at University of Chicago (http://eqtl.uchicago.edu/Home.html),the Genevar (GENe Expression VARiation) at the Wellcome Trust Sanger Institute (http://www.sanger.ac.uk/resources/software/genevar) [42], HaploReg (http://www.broadinstitute.org/mammals/haploreg/haploreg.php) (PMID:22064851), and the GTEx Portal Version 4(http://gtexportal.org/home/) (PMID: 26484569) to investigate whether any of the implicated SNPs may impact the expression of the nearby genes. A cis-eQTL analysis was also performed in TCGA COAD data in 356 Caucasian samples that have demographic and clinical data for 15,008 genes (S1 Text). Third, we analyzed expression data for the implicated genes from 35 pairs of colorectal tumor-normal tissue samples included in the ColoCare Cohort (S2 Text) as well as expression data from the Cancer Genome Atlas (TCGA; http://cancergenome.nih.gov) in 50 pairs of colorectal adenocarcinoma-normal tissue samples. We searched the UCSC Cancer Genomics Browser (https://genome-cancer.ucsc.edu) [37–39] to examine whether the implicated genes showed evidence of differential expression in colorectal tumor tissue and normal tissue. Last, we used the publically available data in the Gene Expression Omnibus site (http://www.ncbi.nlm.nih.gov/geo/) [89, 90] and the gene expression data from normal colon (n = 33) and tumor (n = 28) tissue in the ColoCare Cohort (S2 Text) to investigate whether the expression of implicated genes are correlated with alcohol/smoking history.

### Bioinformatics analysis

We explored potential functional annotations for the SNPs that showed evidence for interactions with either smoking or alcohol in our genome-wide interaction analyses. As detailed in S1 Text, we queried multiple bioinformatics databases using the UCSC genome browser (http://genome.ucsc.edu), HaploReg (http://www.broadinstitute.org/mammals/haploreg/haploreg.php), and literature review of published enhancer signatures of colon cancer.

## Supporting Information

S1 Text Description of study populations included in the Colon Cancer Family Registry (CCFR) and the Genetics and Epidemiology of Colorectal Cancer Consortium (GECCO); functional annotation of identified loci. (DOCX)

S2 Text Description of ColoCare study. (DOCX)

S1 Table Descriptive characteristics for each study included in genome-wide interaction analysis for alcohol consumption. (DOCX)

S2 Table Descriptive characteristics for each study included in genome-wide interaction analysis for smoking. (DOCX)

S3 Table Significant findings for genome-wide interaction analyses with alcohol consumption. (DOCX)

S4 Table Stratification analyses by alcohol consumption for the association of CRC with rs9409565 in men and women and by cancer rite. (DOCX)

S5 Table Interaction between rs9409565 and alcohol consumption for CRC risk based on one reference group and stratified by genotype (last two rows) and by alcohol consumption (last column). (DOCX)

S6 Table Interactions between rs9409565 and alcohol consumption for CRC risk using Empirical Bayesian (EB) interaction analysis, case-control (CC) logistic regression and case-only (CO) interaction analysis. (DOCX)

S7 Table The association between CRC and alcohol consumption stratified by cancer site. (DOCX)

S8 Table Expression Quantitative Trait Locus tagged by rs9409565 for genes in 1Mb. (DOCX)

S1 Fig Association between CRC risk and light/moderate drinker vs non/occasional drinker, stratified by genotype across studies (the interaction estimates and p-values are slightly different from those shown in Table 1 because the Forest plots are based on three separate stratified analyses while results in Table 1 are derived from a single joint effect analysis) (DOCX)

S2 Fig Gene expression levels of *HIATL1* in colorectal tumor tissue and paired adjacent normal tissue from 35 colorectal cancer cases in ColoCare (a,b) and 50 colorectal cancer cases in TCGA (c,d). The 4 probes for *HIATL1* all showed that *HIATL1* expression was significantly higher in tumor tissue than in normal tissue (Paired t test, P = 4.4×10^−9^ to 7.2×10^−5^); the results from two probes that uniquely match *HIATL1* transcript were shown in a (P = 7.2×10^−5^) & b (P = 5.1×10^−7^); These results were replicated in the colorectal tumor-normal-matched samples from TCGA (c,d) (P = 0.025). In figures a, b, and c each line represent a colorectal cancer case connecting the values of gene expression in adjacent normal tissue to tumor tissue from that same case. In figure d the log2 transformed mean expression with 95% confidence interval is shown with a line connecting values of gene expression in tumor and adjacent normal tissue.
(DOCX)

S3 Fig The analysis of variance (ANOVA) to test differences in the expression of *HIATL1* between different levels of lifetime alcohol consumption in 28 colon tumor tissues (a: P value = 0.03; b: P value = 0.30). The lifetime alcohol consumption was categorized into four groups ([0, 4.7), [4.7,12.5), [12.5, 25.3), & > = 25.3 grams of alcohol/day). Y axis: normalized and log2 transformed values of gene expression; X axis: the lifetime alcohol consumption (grams of alcohol/day). Each dot in the figure represented a single sample.
(DOCX)

S4 Fig The analysis of variance (ANOVA) to test differences in the expression of *HIATL1* between different levels of alcohol consumption at reference time (the year before enrollment) in 33 normal colon tissues (a: P value = 0.06; b: P value = 0.07). The alcohol consumption at reference time was categorized into four groups ([0, 4.7), [4.7, 12.5), [12.5, 25.3), & > = 25.3 grams of alcohol/day). Y axis: normalized and log2 transformed values of gene expression; X axis: the lifetime alcohol consumption (grams of alcohol/day). Each dot in the figure represented a single sample. (a) and (b) represented the results from two probes uniquely matching *HIATL1* transcript.
(DOCX)

S5 Fig The associations between rs9409567 and *HIATL1* in eQTL (expression quantitative trait loci) study (Stranger BE, et al. (2012) Patterns of cis regulatory variation in diverse human populations. PLoS Genetics.) among the Utah residents with Northern and Western European ancestry (CEU, n = 109) from Genevar (GENe Expression VARiation) in the Wellcome Trust Sanger Institute. Individual genotypes are plotted on a strip chart, where observed and permuted P values are labeled. r: Spearman's rho; P: observed P value; Pemp: p value of 10,000 permutations.
(DOCX)

S6 Fig Functional annotation of rs9409567 and correlated SNPs in chromosome 9. rs9409565 (shown as green bar) is correlated with 142 variants (r^2^≥0.5 in 1000 Genomes Phase 3 European populations). The tagged variants span across intronic regions and approximately 50kb downstream and 75kb upstream of *HIATL1*. Eigtheen of these variants fall within a transcriptionally active region in colorectal tissue, and four of these variants (rs7042481,rs7858085, rs9409510, rs28406858) are positioned within three variant enhancer loci (VEL, shown as orange bars).
(DOCX)

S7 Fig Functional annotation of rs28406858 in the rs9409567 locus. Rs28406858 is shown as the orange bar and highlighted in blue. The variant is positioned in both a variant enhancer locus in the first intron of *HIATL1* and a protein binding site for ELF1. Bioinformatic annotation suggests this variant is a strong candidate for functional follow-up.
(DOCX)
