# Supplementary material for: Genome-Wide Interaction Analyses between Genetic Variants and Alcohol Consumption and Smoking for Risk of Colorectal Cancer
Source: PLoS Genet. 2016 Oct 10;12(10):e1006296. doi: 10.1371/journal.pgen.1006296 (PMC5065124; doi:10.1371/journal.pgen.1006296)
Supplement: S1 Text — (DOCX) [file pgen.1006296.s001.docx]

**Text S1**

**Description of Study Populations Included in the Colon Cancer Family Registry (CCFR) and the Genetics and Epidemiology of Colorectal Cancer Consortium (GECCO)**

***Ontario Familial Colorectal Cancer Registry*** *(****OFCCR)***. In GECCO, a subset of the Assessment of Risk in Colorectal Tumours in Canada (ARCTIC) from the Ontario Registry for Studies of Familial Colorectal Cancer (OFCCR) was used. Both the case-control study ^1^ and the OFCCR ^2^ have been described in detail previously, as have GWAS results ^3^. In brief, cases were confirmed incident colorectal cancer (CRC) cases ages 20 to 74 years, residents of Ontario identified through comprehensive registry and diagnosed between July 1997 and June 2000. Population-based controls were randomly selected among Ontario residents (random-digit-dialing and listing of all Ontario residents), and matched by sex and 5-year age groups. A total of 1,236 CRC cases and 1,223 controls were successfully genotyped on at least one of the Illumina 1536 GoldenGate assay (Illumina, Inc, San Diego, CA), the Affymetrix GeneChip® Human Mapping 100K and 500K Array Set (Affymetrix, Inc, Santa Clara, CA), and a 10K non-synonymous SNP chip. Analysis was based on a set of unrelated subjects who were non-Hispanic, White by self-report or by investigation of genetic ancestry. We further excluded subjects if there was a sample mix-up, if they were missing epidemiologic questionnaire data, if they were cases with a tumor in the appendix, or if they were overlapped with the Colon Cancer Family Registry. Additionally, only samples genotyped on the Affymetrix GeneChip® 500K Array were utilized in order to avoid coverage issues in imputation.

***The french Association STudy Evaluating RISK for sporadic colorectal cancer (ASTERISK)*** *^4^*. Participants were recruited from the Pays de la Loire region in France between December 2002 and March 2006. Eligibility criteria for cases included being of Caucasian origin, being greater than or 40 years of age at diagnosis, and having no family history of colorectal cancer or polyps. Cases were patients with first primary colorectal cancer diagnosed in one of the six public hospitals and five clinics located in the Pays de la Loire region which participated in the study. Cases were confirmed based on medical and pathology reports. Ccontrols were recruited at two Health Examination Centers of the Pays de la Loire region, and the recruitment of controls greater than or 70 years was completed in the departments of internal medicine and hepatogastroenterology of the University Hospital Center of Nantes, located in the same region. Controls were eligible to participate if they were Caucasian, aged greater than or 40 years, and had no family history of colorectal cancer or polyps. In the presence of the physician, each participant filled out a standardized questionnaire on family information, medical history, lifestyle, and dietary intake. Cases and controls provided a blood sample.

***Colon Cancer Family Registry (CCFR)***. The CCFR is an NCI-supported consortium consisting of six centers dedicated to the establishment of a comprehensive collaborative infrastructure for interdisciplinary studies in the genetic epidemiology of colorectal cancer ^5^. The CCFR includes data from approximately 30,500 total subjects (10,500 probands, and 20,000 unaffected and affected relatives and unrelated controls). Cases and controls, age 20 to 74 years, were recruited at the six participating centers beginning in 1998. CCFR implemented a standardized questionnaire that is administered to all participants, and includes established and suspected risk factors for colorectal cancer, which includes questions on medical history and medication use, reproductive history (for female participants), family history, physical activity, demographics, alcohol and tobacco use, and dietary factors. The Set 1 scan, which has been described previously ^6^, includes population-based cases and age-matched controls from the three population-based centers: Seattle, Toronto and Australia. Cases were genetically enriched by over-sampling those with a young age at onset or positive family history. Controls were matched to cases on age and sex. All cases and controls were self-reported as White, which was confirmed with genotype data. The Set 2 scan includes population-based cases and matched controls from all six Colon CFR centers including Mayo Clinic, Hawaii Cancer Registry, University of Southern California, Fred Hutchinson Cancer Research Center, Cancer Care Ontario and University of Melbourne. As with Set 1, cases were genetically enriched by over-sampling those with a young age at onset or positive family history. Controls were same generation family controls.

***Darmkrebs: Chancen der Verhütung durch Screening (DACHS)*** ^7, 8^.

This German study was initiated as a large population-based case-control study in 2003 in the Rhine-Neckar-Odenwald region (southwest region of Germany) to assess the potential of endoscopic screening for reduction of colorectal cancer risk and to investigate etiologic determinants of disease, particularly lifestyle/environmental factors and genetic factors. Cases with a first diagnosis of invasive colorectal cancer (International Classification of Diseases 10 codes C18-C20) who were at least 30 years of age (no upper age limit), German speaking, a resident in the study region, and mentally and physically able to participate in a one-hour interview, were recruited by their treating physicians either in the hospital a few days after surgery, or by mail after discharge from the hospital. Cases were confirmed based on histologic reports and hospital discharge letters following diagnosis of colorectal cancer. All hospitals treating colorectal cancer patients in the study region participated. Based on estimates from population-based cancer registries, more than 50% of all potentially eligible patients with incident colorectal cancer in the study region were included. Community-based controls were randomly selected from population registries, employing frequency matching with respect to age (5-year groups), sex, and county of residence. Controls with a history of colorectal cancer were excluded. Controls were contacted by mail and follow-up calls. The participation rate was 51%. During an in-person interview, data were collected on demographics, medical history, family history of CRC, and various life-style factors, as were blood and mouthwash samples. This analysis includes participants recruited up to 2010 in this ongoing study, controls were frequency matched to cases on age, gender, and county of residence.

***Diet, Activity, and Lifestyle Study (DALS****) ^9^*. DALS is a population-based case-control study of colon cancer. Participants were recruited between 1991 and 1994 from three locations: the Kaiser Permanente Medical Care Program (KPMCP) of Northern California, an eight-county area in Utah, and the metropolitan Twin Cities area of Minnesota. Eligibility criteria for cases included age at diagnosis between 30 and 79 years, diagnosis with first primary colon cancer (International Classification of Diseases for Oncology-2 codes 18.0 and 18.2-18.9) between October 1^st^ 1991 and September 30^th^ 1994, English speaking, and competency to complete the interview. Individuals with cancer of the rectosigmoid junction or rectum were excluded, as were those with a pathology report noting familial adenomatous polyposis, Crohn’s disease, or ulcerative colitis. A rapid-reporting system was used to identify all incident cases of colon cancer resulting in the majority of cases being interviewed within four months of diagnosis. Controls from KPMCP were randomly selected from membership lists. In Utah, controls under 65 years of age were randomly selected through random-digit dialing and driver license lists. Controls, 65 years of age and older, were randomly selected from Health Care Financing Administration lists. In Minnesota, controls were identified from Minnesota driver’s license or state ID lists. Controls were matched to cases by 5-year age groups and sex. The Set I scan consisted of a subset of the study designed above, from Utah, Minnesota, and KPMCP, and was restricted to subjects who self-reported as White non-Hispanic. The Set 2 scan consisted of subjects from Utah and Minnesota that were not genotyped in Set 1. Set 2 was restricted to subjects who self-reported as White non-Hispanic and those that had appropriate consent to post data to dbGaP.

***Hawai’i Colorectal Cancer Studies 2 & 3 (Colo2&3)*** ^10^. Patients with colorectal cancer were identified through the rapid reporting system of the Hawaii SEER registry and consisted of all Japanese, Caucasian, and Native Hawaiian residents of Oahu who were newly diagnosed with an adenocarcinoma of the colon or rectum between January 1994 and August 1998. Control subjects were selected from participants in an on-going population-based health survey conducted by the Hawaii State Department of Health and from Health Care Financing Administration participants. Controls were matched to cases by sex, ethnicity, and age (within two years). Personal interviews were obtained from 768 matched pairs, resulting in a participation rate of 58.2% for cases and 53.2% for controls. A questionnaire, administered during an in-person interview, included questions about demographics, lifetime history of tobacco, alcohol use, aspirin use, physical activity, personal medical history, family history of colorectal cancer, height and weight, diet (Food Frequency Questionnaire), and postmenopausal hormone use. A blood sample was obtained from 548 (71%) of interviewed cases and 662 (86%) of interviewed controls. SEER staging information was extracted from the Hawaii Tumor Registry. In GECCO, self-reported Caucasian subjects with DNA, and clinical and epidemiologic data were selected for genotyping.

***Health Professionals Follow-up Study (HPFS)*** ^11^. The HPFS is a parallel prospective study to the Nurses’ Health Study (NHS). The HPFS cohort comprises 51,529 men who, in 1986, responded to a mailed questionnaire. The participants are U.S. male dentists, optometrists, osteopaths, podiatrists, pharmacists, and veterinarians born between 1910 and 1946. Participants have provided information on health related exposures, including: current and past smoking history, age, weight, height, diet, physical activity, aspirin use, and family history of colorectal cancer. Colorectal cancer and other outcomes were reported by participants or next-of-kin and followed up through review of the medical and pathology record by physicians. Overall, more than 97% of self-reported colorectal cancers were confirmed by medical record review. Information was abstracted on histology and primary location. Incident cases are defined as those occurring after the subject provided the blood sample. Prevalent cases are defined as those occurring after enrollment in the study, but prior to the subject providing the blood sample. Follow-up has been excellent, with 94% of the men responding to date. Colorectal cancer cases were ascertained through January 1, 2008. In 1993-95, 18,825 men in HPFS mailed in blood samples by overnight courier which were aliquoted into buffy coat and stored in liquid nitrogen. In 2001-04, 13,956 men in HPFS who had not previously provided a blood sample mailed in a "swish-and-spit" sample of buccal cells. Incident cases are defined as those occurring after the subject provided a blood or buccal sample. Prevalent cases are defined as those occurring after enrollment in the study in 1986, but prior to the subject providing either a blood or buccal sample. After excluding participants with histories of cancer (except non-melanoma skin), ulcerative colitis, or familial polyposis, two case-control sets were constructed from which DNA was isolated from either buffy coat or buccal cells for genotyping: 1) a case-control set with cases of colorectal cancer matched to randomly selected controls who provided a blood sample and were free of colorectal cancer at the same time the colorectal cancer was diagnosed in the cases; 2) a case-control set with cases of colorectal cancer matched to randomly selected controls who provided a buccal sample and were free of colorectal cancer at the same time the colorectal cancer was diagnosed in the case. For both case-control sets, matching criteria included year of birth (within 1 year) and month/year of blood or buccal cell sampling (within six months). Cases were pair matched 1:1, 1:2, or 1:3 with a control participant(s).

In addition to colorectal cancer cases and controls, a set of adenoma cases and matched controls with available DNA from buffy coat were selected for genotyping. Over follow-up, data were collected on endoscopic screening practices and, if individuals have been diagnosed with polyp, the polyps were confirmed to be adenomatous by medical record review. Adenoma cases were ascertained through January 1, 2008. A separate case-control set was constructed of participants diagnosed with advanced adenoma matched to control participants who underwent a lower endoscopy in the same time period and did not have an adenoma. Advanced adenoma was defined as an adenoma >=1 cm in diameter and / or with tubulovillous, villous, or high-grade dysplasia / carcinoma-in-situ histology. Matching criteria included year of birth (within one year) and month/year of blood sampling (within six months), the reason for their lower endoscopy (screening, family history, or symptoms) and the time period of any prior endoscopy (within two years). Controls matched to cases with a distal adenoma either had a negative sigmoidoscopy or colonoscopy exam and controls matched to cases with proximal adenoma all had a negative colonoscopy.

***Multiethnic Cohort Study (MEC)*** ^12^. MEC was initiated in 1993 to investigate the impact of dietary and environmental factors on major chronic diseases, particularly cancer, in ethnically diverse populations in Hawai’i and California. The study recruited 96,810 men and 118,441 women aged 45 to 75 years between 1993 and 1996. Incident colorectal cancer cases occurring since January 1995, and controls were contacted for blood or saliva samples. The median interval between diagnosis and blood draw was 14 months (interquartile range, 10-19) among cases and the participation rate 74%. A sample of cohort participants was randomly selected to serve as controls at the onset of the nested case-control study (participation rate 66%). The selection was stratified by sex, age, and race/ethnicity. Colorectal cancer cases are identified through the Rapid Reporting System of the Hawai’i Tumor Registry and through quarterly linkage to the Los Angeles County Cancer Surveillance Program. Both registries are members of SEER. In GECCO, self-reported White subjects from the nested case-control study described above with DNA, and clinical and epidemiologic data were selected for genotyping

***Nurses’ Health Study (NHS)*** ^13^. The NHS cohort began in 1976 when 121,700 married female registered nurses aged 30 to 55 years returned the initial questionnaire that ascertained a variety of important health-related exposures. Since 1976, follow-up questionnaires have been mailed every two years. Colorectal cancer and other outcomes were reported by participants or next-of-kin and followed up through review of the medical and pathology record by physicians. Overall, more than 97% of self-reported colorectal cancers were confirmed by medical-record review. Information was abstracted on histology and primary location. Follow-up has been high: as a proportion of the total possible follow-up time, follow-up has been over 92%. Colorectal cancer cases were ascertained through June 1, 2008. In 1989-90, 32,826 women in NHS I, mailed in blood samples by overnight courier which were aliquoted into buffy coat and stored in liquid nitrogen. In 2001-04, 29,684 women in NHS I who did not previously provide a blood sample mailed in a "swish-and-spit" sample of buccal cells. Incident cases are defined as those occurring after the subject provided a blood or buccal sample. Prevalent cases are defined as those occurring after enrollment in the study in 1976, but prior to the subject providing either a blood or buccal sample. After excluding participants with histories of cancer (except non-melanoma skin), ulcerative colitis, or familial polyposis, two case-control sets were constructed from which DNA was isolated from either buffy coat or buccal cells for genotyping: 1) a case-control set with cases of colorectal cancer matched to randomly selected controls who provided a blood sample and were free of colorectal cancer at the same time the colorectal cancer was diagnosed in the case; 2) a case-control set with cases of colorectal cancer matched to randomly selected controls who provided a buccal sample and were free of colorectal cancer at the same time the colorectal cancer was diagnosed in the cases. For both case-control sets, matching criteria included year of birth (within one year) and month / year of blood or buccal cell sampling (within six months). Cases were pair matched 1:1, 1:2, or 1:3 with a control participant(s).

In addition to colorectal cancer cases and controls, a set of adenoma cases and matched controls with available DNA from buffy coat were selected for genotyping. Over follow-up, data were collected on endoscopic screening practices and, if individuals have been diagnosed with polyp, the polyps confirmed to be adenomatous by medical record review. Adenoma cases were ascertained through June 1, 2008. A separate case-control set was constructed of participants diagnosed with advanced adenoma matched to control participants who underwent a lower endoscopy in the same time period and did not have an adenoma. Advanced adenoma was defined as an adenoma > 1 cm in diameter and / or with tubulovillous, villous, or high-grade dysplasia / carcinoma-in-situ histology. Matching criteria included year of birth (within one year) and month/year of blood sampling (within six months), the reason for their lower endoscopy (screening, family history, or symptoms) and the time period of any prior endoscopy (within two years). Controls matched to cases with a distal adenoma either had a negative sigmoidoscopy or colonoscopy exam and controls matched to cases with proximal adenoma all had a negative colonoscopy.

***Physician’s Health Study (PHS****)* ^14, 15^. The PHS was established as a randomized, double-blind, placebo-controlled trial of aspirin and ß-carotene among 22,071 healthy U.S. male physicians, between 40 and 84 years of age in 1982. Participants completed two mailed questionnaires before being randomly assigned, additional questionnaires at six and 12 months, and questionnaires annually thereafter. In addition, participants were sent postcards at six months to ascertain status. From August 1982 to December 1984, 14,916 baseline blood samples were collected from the physicians during the run-in phase before randomization. When participants report a diagnosis of cancer, medical records and pathology reports are reviewed by study physicians who are blinded to exposure data. Among those who provided baseline blood samples, colorectal cases were ascertained through March 31, 2008, and controls were matched on age (within one year for younger participants, up to five years for older participants) and smoking status (never, past, current). Cases were “pair” matched 1:1, 1:2 or 1:3 with a control participant(s). Due to DNA availability samples were genotyped in two batches on the same platform at the same genotyping center at different time points.

***Prostate, Lung, Colorectal, and Ovarian Cancer Screening Trial (PLCO)***. PLCO enrolled 154,934 participants (men and women, aged between 55 and 74 years) at ten centers into a large, randomized, two-arm trial to determine the effectiveness of screening to reduce cancer mortality. Sequential blood samples were collected from participants assigned to the screening arm. Participation was 93% at the baseline blood draw. In the observational (control) arm, buccal cells were collected via mail using the “swish-and-spit” protocol and participation rate was 65%. Details of this study have been previously described ^16, 17^ and are available online (http://dcp.cancer.gov/plco).

The Set 1 scan included a subset of 577 colon cancer cases self-reported as being non-Hispanic White with available DNA samples, questionnaire data, and appropriate consent for ancillary epidemiologic studies. Cases were excluded if they had a history of inflammatory bowel disease, polyps, polyposis syndrome or cancer (excluding basal or squamous cell skin cancer). Controls come from the Cancer Genetic Markers of Susceptibility (CGEMS) prostate cancer scan ^18, 19^ (all male) and the GWAS of Lung Cancer and Smoking ^20^ (enriched for smokers) along with an additional 92 non-Hispanic White female controls. For the Set 2 scan, cases were colorectal cancers from both arms of the trial, which were not already included in Set 1. Samples were excluded if participants did not sign appropriate consents, if DNA was unavailable, if baseline questionnaire data with follow-up were unavailable, if they had a history of colon cancer prior to the trial, if they were a rare cancer, and if they were already in colon GWAS, or if they were a control in the prostate or lung populations. Controls were frequency matched 1:1 to cases without replacement, and cases were not eligible to be controls. Matching criteria were age at enrollment (two year blocks), enrollment date (two year blocks), sex, race / ethnicity, trial arm, and study year of diagnosis (i.e. controls must be cancer free into the case's year of diagnosis).

***Postmenopausal Hormones Supplementary Study to the Colon Cancer Family Registry (PMH-CCFR*)** ^21^. Eligible case patients included all female residents, ages 50 to 74 years, residing in the 13 counties in Washington State reporting to the Cancer Surveillance SEER program, who were newly diagnosed with invasive colorectal adenocarcinoma (ICD-O C18.0, C18.2-.9, C19.9, C20.0-.9) between October 1998 and February 2002. Eligibility for all individuals was limited to those who were English-speaking with available telephone numbers, in which they could be contacted. On average, cases were identified within four months of diagnosis. The overall response proportion of eligible cases identified was 73%. Community-based controls were randomly selected according to age distribution (in 5-year age intervals) of the eligible cases by using lists of licensed drivers from the Washington State Department of Licensing for individuals, ages 50 to 64 years, and rosters from the Health Care Financing Administration (now the Centers for Medicare and Medicaid) for individuals older than 64 years. The overall response proportion of eligible controls was 66%. In GECCO, samples with sufficient DNA extracted from blood were genotyped. Only participants that were not part of the CCFR Seattle site were included in the sample set.

***VITamins And Lifestyle (VITAL).*** The VITamins And Lifestyle (VITAL) cohort comprises of 77,721 Washington State men and women aged 50 to 76 years, recruited from 2000 to 2002 to investigate the association of supplement use and lifestyle factors with cancer risk. Subjects were recruited by mail, from October 2000 to December 2002, using names purchased from a commercial mailing list. All subjects competed a 24 page questionnaire and buccal-cell specimens for DNA were self-collected by 70% of the participants. Subjects are followed for cancer by linkage to the western Washington SEER cancer registry and are censored when they move out of the area covered by the registry or at time of death. Details of this study have been previously described ^22^. In GECCO, a nested case-control set was genotyped. Samples included, colorectal cancer cases with DNA, excluding subject with colorectal cancer before baseline, in situ cases, (large cell) neuroendocrine carcinoma, squamous cell carcinoma, carcinoid tumor, Goblet cell carcinoid, any type of lymphoma, including non-Hodgkin, Mantle cell, large B-cell, or follicular lymphoma. Controls were matched on age at enrollment (within one year), enrollment date (within one year), sex, and race / ethnicity. One control was randomly selected per case among all controls that matched on the four factors above and where the control follow-up time was greater than follow-up time of the case until diagnosis.

**Women’s Health Initiative (WHI).** WHI is a long-term health study of 161,808 post-menopausal women aged 50 to 79 years at 40 clinical centers throughout the U.S. WHI comprises a Clinical Trial (CT) arm, an Observational Study (OS) arm, and several extension studies. The details of WHI have been previously described ^23, 24^ and are available online (https://cleo.whi.org/SitePages/Home.aspx). In GECCO, Set 1 cases were selected from the September 12, 2005 database and were comprised of centrally adjudicated colon cancer cases from the Observational Study (OS) who self-reported as White. Controls were first selected among controls previously genotyped as part of a Hip Fracture GWAS conducted within the WHI OS and matched to cases on age (within three years) enrollment date (within 365 days), hysterectomy status, and prevalent conditions at baseline. For 37 cases, there was not a control match in the Hip Fracture GWAS. For these participants, we identified a matched control in the WHI OS based on same criteria. In the Set 2 scan, cases were selected from the August 2009 database and were comprised of centrally adjudicated colon and colorectal cancer cases from the OS and CT who were not genotyped in Set 1. In addition, case and control participants were subject to the following exclusion criteria: a prior history of colorectal cancer at baseline, IRB approval not available for data submission into dbGaP, and not sufficient DNA available. Matching criteria included age (within years), race/ethnicity, WHI date (within three years), WHI Calcium and Vitamin D study date (within three years), and randomization arms (OS flag, hormone therapy assignments, dietary modification assignments, calcium/vitamin D assignments). In addition, they were matched on the four regions of randomization centers. Each case was matched with one control (1:1) that exactly met the matching criteria. Control selection was done in a time-forward manner, selecting one control for each case first from the risk set at the time of the case’s event. The matching algorithm was allowed to select the closest match based on a criterion to minimize an overall distance measure ^25^. Each matching factor was given the same weight. Additional available controls that were genotyped as part of the Hip Fracture GWAS were included to improve power.

**Functional Annotation of Identified Loci**

To prioritize likely causal variants and generate testable functional hypotheses about the underlying mechanisms of SNP-environment associations for colorectal cancer risk, the bioinformatics framework described below was used to query publicly available variant annotation datasets. Several databases are available for the functional characterization of putative disease causing loci such as HaploReg ^26^ (maintained by the Broad Institute), the University of California, Santa Cruz (UCSC) genome browser ^27^, and the NHLBI exome variant server (EVS).

Although SNPs in high LD with the most significant interaction SNPs were often included in tests of association, frequentist testing is not reflective of actual function. As such, the underlying functional interaction SNP may not be the most significant association. Therefore, functional hypotheses were made for (1) the most significant SNP-environment association with colorectal cancer and (2) all variants tagged by the most significant SNP in the interaction (LD r^2^>0.5 in EUR (CEU+FIN+GBR+IBS+TSI) from phase 3 1000 Genomes Project). This SNP list was generated using the University of Southern California (USC) Norris Bioinformatics Core resource rAggr (http://raggr.usc.edu/). While each putative exonic variant would have been evaluated for potential deleteriousness using annotations such as PolyPhen2, Combined Annoation Dependent Depletion (CADD^26^-http://cadd.gs.washington.edu/), or Deleterious Annotation of genetic variants using Neural Networks (DANN^27^ - https://cbcl.ics.uci.edu/public_data/DANN/) none of the variants were positioned in the coding region. As such each non-coding variant was annotated for regulatory evidence consistent with enhancers, promoters, insulators, silencers, and other effects related to gene expression.

Annotation of non-protein-coding regions operates under the hypothesis that trait-associated alleles exert their effects by influencing transcript levels through multiple regulatory mechanisms. Here we assume that interaction SNPs similarly exert there effects by influencing the expression of target genes that modify environmental factors for CRC risk (alcohol/smoking). HaploReg is useful for an initial survey of non-coding genome variants i for regulatory evidence such as DNaseI Hypersensitivity (DHS), histone modifications, expression Quantitative Loci (QTL)s, transcription factor binding sites, protein- binding motif analysis, and evolutionary conservationThis database explores LD block SNP lists using chromatin state and protein binding annotations from ENCODE and NIH Roadmap Epigenomics projects. After establishing putative target genes for the locus through eQTL analysis, as well as tissues and transcription factors of interest, The UCSC genome browser was used for a deeper interrogation of potential functional variants using both publicly available datasets , as well and CR and CRC specific enhancer annotations (Histone Modificaton - H3K27ac)^28^.

Since distal enhancers often facilitate cell-type specific expression, it is helpful to look for evidence in a variety of cell lines and tissues in addition to those directly related to CRC. Furthermore, the comparison of chromatin states between CRC cancer cell lines to phenotypically normal CR tissue can be used to identify gain and loss of enhancer activity that may promote colon carcinogenesis. Variant chromatin remodeling and histone modification can provide additional evidence for cell-specific regulatory elements. To identify variant chromatin state loci the “Histone Modification” tracks within the ENCODE Analysis Hub (http://ftp.ebi.ac.uk/pub/databases/ensembl/encode/integration_data_jan2011/hub.txt) and “Uniformly Signal” within the Roadmap Epigenomics Data Complete Collection at Wash U VizHub (http://vizhub.wustl.edu/VizHub/RoadmapReleaseAll.txt ), was used to identify variants from our SNP list that were positioned in putative regulatory elements demarked by a core histone mark (H3K4me3, H3K4me1, H3K36me3, H3K27me3, H3K9me3). Of particular interest, the gain and loss of the H3k27ac histone mark is thought to identify active enhancers and is enriched in GWAS loci. Although a large number of cell lines and tissues were queried to assess cell-type specific regulation, CACO2 and HCT116 (colon cancer cell lines) from ENCODE assays and normal colon and rectal tissue (n=3) from Roadmap assays were of particular interest for colon cancer susceptibility loci.

Transcription factor binding sites (TFBS) are susceptible to DNase I cutting and ENCODE has assayed hypersensitivity in a large collection of cell types. DNase I hypersensitivity is a more precise demarcation of TFBS than chromatin methylation and acetylation patterns detected by ChIP-seq. As such, the DNase I hypersensitivity track was used to provide a more specific demarcation of open chromatin within broader histone modification signals. Although we have prioritized variants positioned within both a DHS peak and a histone modification mark, it remains possible for regulatory variants to exert their effects through nucleosome positioning and as such all variants positioned within the ChIP-seq signal would remain of interest for laboratory follow-up.

The ChIP-Seq TFBS track provides evidence for the binding of specific proteins, providing a strong mechanistic hypothesis for laboratory follow-up. However, most transcription factors have yet to be comprehensively assayed across tissues and cell-types. Though less specific than ChIP-Seq methodology, JASPAR, ConSite and HaploReg PWM databases were used to query a larger number of conserved TFBS to predict alterations in conserved binding motifs between reference and alternate alleles. After identification of proteins that bind to a particular region through ChIP-seq, these PWM libraries can be particularly useful for generating hypotheses about perturbation of protein binding.

In addition to the cell-type/tissue specific chromatin state maps provided through Roadmap Epigenomics and ENCODE we used active enhancer mapping previously published by Akhtar-Zaidi et al to analyze gain and loss of enhancer activity genome wide in primary colon cancer lines relative to normal colon crypts referred to as variant enhancer loci (VEL). Enhancers were mapped in 3 normal crypts and 6 primary colon cancer cell lines using the H3K27ac marks.

Variants were prioritized for functional follow-up if they were positioned in 1) SNPs in Colorectal VEL or 2) differential regulatory elements/protein binding sites from cell-types tissues implicated through the eQTL analysis.

Reference List

(1) Cotterchio M, Manno M, Klar N, McLaughlin J, Gallinger S. Colorectal screening is associated with reduced colorectal cancer risk: a case-control study within the population-based Ontario Familial Colorectal Cancer Registry. *Cancer Causes Control* 2005;16(7):865-875.

(2) Cotterchio M, Keown-Eyssen G, Sutherland H et al. Ontario familial colon cancer registry: methods and first-year response rates. *Chronic Dis Can* 2000;21(2):81-86.

(3) Zanke BW, Greenwood CM, Rangrej J et al. Genome-wide association scan identifies a colorectal cancer susceptibility locus on chromosome 8q24. *Nat Genet* 2007;39(8):989-994.

(4) Küry S, Buecher B, Robiou-du-Pont S et al. Combinations of cytochrome P450 gene polymorphisms enhancing the risk for sporadic colorectal cancer related to red meat consumption. *Cancer Epidemiol Biomarkers Prev* 2007;16(7):1460-1467.

(5) Newcomb PA, Baron J, Cotterchio M et al. Colon Cancer Family Registry: an international resource for studies of the genetic epidemiology of colon cancer. *Cancer Epidemiol Biomarkers Prev* 2007;16(11):2331-2343.

(6) Figueiredo JC, Lewinger JP, Song C et al. Genotype-environment interactions in microsatellite stable/microsatellite instability-low colorectal cancer: results from a genome-wide association study. *Cancer Epidemiol Biomarkers Prev* 2011;20(5):758-766.

(7) Brenner H, Chang-Claude J, Seiler CM, Rickert A, Hoffmeister M. Protection from colorectal cancer after colonoscopy: population-based case-control study. *Ann Intern Med* 2011;154(1):22-30.

(8) Lilla C, Verla-Tebit E, Risch A et al. Effect of NAT1 and NAT2 genetic polymorphisms on colorectal cancer risk associated with exposure to tobacco smoke and meat consumption. *Cancer Epidemiol Biomarkers Prev* 2006;15(1):99-107.

(9) Slattery ML, Potter J, Caan B et al. Energy balance and colon cancer--beyond physical activity. *Cancer Res* 1997;57(1):75-80.

(10) Le Marchand L, Hankin JH, Wilkens LR et al. Combined effects of well-done red meat, smoking, and rapid N-acetyltransferase 2 and CYP1A2 phenotypes in increasing colorectal cancer risk. *Cancer Epidemiol Biomarkers Prev* 2001;10(12):1259-1266.

(11) Rimm EB, Stampfer MJ, Colditz GA, Chute CG, Litin LB, Willett WC. Validity of self-reported waist and hip circumferences in men and women. *Epidemiology* 1990;1(6):466-473.

(12) Kolonel LN, Henderson BE, Hankin JH et al. A multiethnic cohort in Hawaii and Los Angeles: baseline characteristics. *Am J Epidemiol* 2000;151(4):346-357.

(13) Belanger CF, Hennekens CH, Rosner B, Speizer FE. The Nurses' Health Study. *Am J Nurs* 1978;78(6):1039-1040.

(14) Hennekens CH, Eberlein K. A randomized trial of aspirin and beta-carotene among U.S. physicians. *Prev Med* 1985;14(2):165-168.

(15) Christen WG, Gaziano JM, Hennekens CH. Design of Physicians' Health Study II--a randomized trial of beta-carotene, vitamins E and C, and multivitamins, in prevention of cancer, cardiovascular disease, and eye disease, and review of results of completed trials. *Ann Epidemiol* 2000;10(2):125-134.

(16) Prorok PC, Andriole GL, Bresalier RS et al. Design of the Prostate, Lung, Colorectal and Ovarian (PLCO) Cancer Screening Trial. *Control Clin Trials* 2000;21(6 Suppl):273S-309S.

(17) Gohagan JK, Prorok PC, Hayes RB, Kramer BS. The Prostate, Lung, Colorectal and Ovarian (PLCO) Cancer Screening Trial of the National Cancer Institute: history, organization, and status. *Control Clin Trials* 2000;21(6 Suppl):251S-272S.

(18) National Cancer Institute. Cancer Genetic Markers of Susceptibility (CGEMS) data website. 2009.

Ref Type: Online Source

(19) Yeager M, Chatterjee N, Ciampa J et al. Identification of a new prostate cancer susceptibility locus on chromosome 8q24. *Nat Genet* 2009.

(20) Landi MT, Chatterjee N, Yu K et al. A genome-wide association study of lung cancer identifies a region of chromosome 5p15 associated with risk for adenocarcinoma. *Am J Hum Genet* 2009;85(5):679-691.

(21) Newcomb PA, Zheng Y, Chia VM et al. Estrogen plus progestin use, microsatellite instability, and the risk of colorectal cancer in women. *Cancer Res* 2007;67(15):7534-7539.

(22) White E, Patterson RE, Kristal AR et al. VITamins And Lifestyle cohort study: study design and characteristics of supplement users. *Am J Epidemiol* 2004;159(1):83-93.

(23) Hays J, Hunt JR, Hubbell FA et al. The Women's Health Initiative recruitment methods and results. *Ann Epidemiol* 2003;13(9 Suppl):S18-S77.

(24) The Women's Health Initiative Study Group. Design of the Women's Health Initiative clinical trial and observational study. *Control Clin Trials* 1998;19(1):61-109.

(25) Bergstralh EJ, Kosanke JL. Computerized matching of cases to controls. Department of Health Sciences Research, Mayo Clinic, Rochester MN; 1995. Report No.: 56.

(26) Kircher M, Witten DM, Jain P, O'Roak BJ, Cooper GM, Shendure J. A general framework for estimating the relative pathogenicity of human genetic variants. *Nat Genet* 2014;46(3):310-315.

(27) Quang D, Chen Y, Xie X. DANN: a deep learning approach for annotating the pathogenicity of genetic variants. *Bioinformatics* 2015;31(5):761-763.

(28) Akhtar-Zaidi B, Cowper-Sal-lari R, Corradin O et al. Epigenomic enhancer profiling defines a signature of colon cancer. *Science* 2012;336(6082):736-739.
