# Supplementary material for: Genome-Wide Interaction Analyses between Genetic Variants and Alcohol Consumption and Smoking for Risk of Colorectal Cancer
Source: PLoS Genet. 2016 Oct 10;12(10):e1006296. doi: 10.1371/journal.pgen.1006296 (PMC5065124; doi:10.1371/journal.pgen.1006296)
Supplement: S2 Text — (DOCX) [file pgen.1006296.s002.docx]

**Text S2**

**Description of ColoCare Study:**

The ColoCare Study (<http://www.dkfz.de/en/praeventive-onkologie/colocare.html>) is a prospective cohort study of colorectal cancer (CRC) patients, which started in October 2010 at the Division of Preventive Oncology, National Center for Tumor Diseases, Heidelberg, Germany. The ColoCare Study investigates predictors of cancer recurrence, survival, health-related quality-of-life and treatment toxicities. Patients are recruited at time of first diagnosis. RNA was extracted from ~50mg tissue (fresh frozen) using the QIAGEN AllPrep DNA/RNA Mini Kit. Gene expression patterns were measured using Illumina HumanHT-12 Expression BeadChips that target more than 47,000 transcripts including known splice variants across the human transcriptome. In this study, (1) we evaluated the expression levels of *HIATIL1* and *CNTN5* genes between tumor tissues and adjacent normal tissues from 35 colorectal cancer cases; (2) for examining the effects of alcohol consumption on gene expression, we mined data from the expression of *HIATIL1* and *CNTN5* genes in n=28 tumor and n=33 tumor-distant tissue samples (normal mucosa) of CRC patients (among them, 23 are paired). Gene expression levels for each sample are determined using Illumina GenomeStudio software (Illumina, www.illumina.com). The statistical analysis was performed using gene expression data with log2 transformation followed by a robust spline normalization. Paired t test was performed for (1) to examine the difference in gene expression between colorectal tumor and adjacent normal tissues; Analysis of Variance (ANOVA) was conducted for (2) to test the difference in the gene expression levels across different alcohol consumption levels.
