# Supplementary material for: Genome-Wide Interaction Analyses between Genetic Variants and Alcohol Consumption and Smoking for Risk of Colorectal Cancer
Source: PLoS Genet. 2016 Oct 10;12(10):e1006296. doi: 10.1371/journal.pgen.1006296 (PMC5065124; doi:10.1371/journal.pgen.1006296)
Supplement: S1 Table — (DOCX) [file pgen.1006296.s003.docx]

**S1 Table. Descriptive characteristics for each study included in genome-wide interaction analysis for alcohol consumption.**

| **Study ^a^** | **Design** | **Men** | | | | | |  | **Women** | | | | | |
| --- | --- | --- | --- | --- | --- | --- | --- | --- | --- | --- | --- | --- | --- | --- |
|  |  | **Cases, n** | **Controls, n** | **Age, years** | **Alcohol Consumption ^b^, n(%)** | | |  | **Cases, n** | **Controls,**  **n** | **Age, years** | **Alcohol Consumption ^b^, n (%)** | | |
|  |  |  |  | **Mean (SD)** | **Non-/occasional drinkers** | **Light-to-moderate drinkers** | **Heavy drinkers** |  |  |  | **Mean (SD)** | **Non-/occasional drinker** | **Light-to-moderate drinker** | **Heavy drinker** |
| Colo23 | case-control | 47 | 68 | 65 (11) | 47 (41) | 54 (47) | 14 (12) |  | 40 | 54 | 66 (11) | 39 (41) | 42 (45) | 13 (14) |
| DACHS | case-control | 1397 | 1321 | 69 (10) | 483 (19) | 1518 (55) | 717 (26) |  | 949 | 832 | 70 (11) | 795 (43) | 902 (52) | 84 (5) |
| DALS | case-control | 618 | 643 | 64 (9) | 557 (46) | 485 (37) | 219 (17) |  | 498 | 531 | 64 (10) | 677 (67) | 307 (29) | 45 (4) |
| HPFS | cohort | 359 | 357 | 65 (9) | 173 (24) | 428 (59) | 115 (17) |  | - | - | - | - | - | - |
| HPFS_AD | cohort | 299 | 329 | 61 (8) | 160 (25) | 385 (61) | 83 (13) |  | - | - | - | - | - | - |
| MEC | case-control | 166 | 177 | 63 (8) | 104 (30) | 143 (42) | 96 (28) |  | 148 | 157 | 63 (8) | 139 (46) | 119 (39) | 47 (15) |
| NHS | cohort | - | - | - | - | - | - |  | 490 | 565 | 57 (7) | 489 (46) | 520 (49) | 46 (4) |
| NHS_AD | cohort | - | - | - | - | - | - |  | 498 | 855 | 60 (7) | 658 (49) | 613 (44) | 82 (6) |
| PHS | cohort | 375 | 386 | 59 (9) | 160 (21) | 570 (75) | 31 (4) |  | - | - | - | - | - | - |
| PLCO | cohort | 252 | 428 | 65 (5) | 250 (37) | 301 (44) | 129 (19) |  | 182 | 256 | 64 (5) | 218 (50) | 190 (43) | 30 (7) |
| VITAL | cohort | 147 | 145 | 66 (6) | 105 (36) | 138 (47) | 49 (17) |  | 132 | 134 | 67 (6) | 136 (51) | 118 (44) | 12 (5) |
| WHI | cohort | - | - | - | - | - | - |  | 1461 | 1527 | 67 (7) | 1650 (55) | 1197 (40) | 141 (5) |

^a^: Colon23: Hawaii Colorectal Cancer Studies 2 and 3 ;DACHS: Darmkrebs: Chancen der Verhütung durch Screening; DALS: Diet, Activity and Lifestyle Study; HPFS: Health Professionals Follow-up Study; HPFS_AD: Health Professionals Follow-up Study for colorectal adenoma ; MEC: Multiethnic Cohort Study; NHS: Nurses’ Health Study; NHS_AD: Nurses’ Health Study for colorectal adenoma; PHS: Physicians’ Health Study; PLCO: Prostate, Lung, Colorectal and Ovarian Cancer; Screening Trial; VITAL: VITamins And Lifestyle; WHI: Women’s Health Initiative.

^b^: non-/occasional drinkers: drinking < 1 gram of alcohol per day); light-to-moderate drinkers: drinking 1-28 grams of alcohol per day; heavy drinkers: drinking >28 grams of alcohol per day.
