# Supplementary material for: Genome-Wide Interaction Analyses between Genetic Variants and Alcohol Consumption and Smoking for Risk of Colorectal Cancer
Source: PLoS Genet. 2016 Oct 10;12(10):e1006296. doi: 10.1371/journal.pgen.1006296 (PMC5065124; doi:10.1371/journal.pgen.1006296)
Supplement: S2 Table — (DOCX) [file pgen.1006296.s004.docx]

**S2 Table: Descriptive characteristics for each study included in genome-wide interaction analysis for smoking.**

| **Study ^a^** | **Design** | **Men** | | | | |  | **Women** | | | | |
| --- | --- | --- | --- | --- | --- | --- | --- | --- | --- | --- | --- | --- |
|  |  | **Cases** | **Controls** | **Age, years** | **Ever Smoker** | **Pack-years** |  | **Cases** | **Controls** | **Age, years** | **Ever Smoker** | **Pack-years** |
|  |  | **n** | **n** | **Mean (SD)** | **n (%)** | **Mean (SD)** |  | **n** | **n** | **Mean (SD)** | **n (%)** | **Mean (SD)** |
| ASTERISK | case-control | 538 | 522 | 65 (10) | 622 (59) | - |  | 331 | 420 | 66 (11) | 105 (14) | - |
| CCFR ^b^ | case-control | 602 | 467 | 55 (11) | 687 (64) | 26.6 (22.1) |  | 558 | 509 | 54 (12) | 530 (50) | 19.4 (19.0) |
| Colo23 | case-control | 47 | 70 | 65 (11) | 66 (56) | 35.0 (30.2) |  | 40 | 55 | 66 (11) | 50 (53) | 22.8 (20.8) |
| DACHS | case-control | 1406 | 1340 | 68 (10) | 1960 (71) | 19.3 (18.7) |  | 967 | 862 | 69 (11) | 684 (37) | 13.1 (15.4) |
| DALS | case-control | 617 | 643 | 68 (10) | 811 (64) | 37.9 (28.7) |  | 496 | 531 | 64 (10) | 424 (41) | 28.8 (23.7) |
| HPFS | cohort | 384 | 379 | 65 (9) | 437 (57) | 20.0 (21.3) |  | - | - | - | - | - |
| HPFS_AD | cohort | 297 | 334 | 61 (9) | 311 (49) | 19.6 (22.3) |  | - | - | - | - | - |
| MEC | case-control | 177 | 183 | 63 (8) | 245 (68) | 31.0 (20.4) |  | 150 | 161 | 63 (8) | 190 (61) | 24.5 (17.2) |
| NHS | cohort | - | - | - | - | - |  | 545 | 949 | 60 (7) | 854 (57) | 25.2 (20.9) |
| NHS_AD | cohort | - | - | - | - | - |  | 512 | 576 | 57 (7) | 588 (54) | 21.8 (18.9) |
| OFCCR | case-control | 200 | 292 | 62 (8) | 332 (68) | 28.2 (21.2) |  | 355 | 225 | 63 (8) | 283 (49) | 21.3 (18.2) |
| PLCO | cohort | 565 | 561 | 64 (5) | 696 (62) | 40.8 (30.1) |  | 426 | 376 | 64 (5) | 367 (46) | 30.1 (24.1) |
| PMH-CCFR | case-control | - | - | - | - | - |  | 280 | 122 | 63 (7) | 206 (51) | 23.8 (22.4) |
| VITAL | cohort | 149 | 149 | 66 (6) | 197 (66) | 33.6 (26.7) |  | 133 | 138 | 67 (6) | 141 (52) | 24.2 (21.1) |
| WHI | cohort | - | - | - | - | - |  | 1444 | 1518 | 66 (6) | 1493 (50) | 24.6 (26.2) |

^a^: ASTERISK: The French Association STudy Evaluating RISK for sporadic colorectal cancer; CCFR: Colon Cancer Family Registry; Colon23: Hawaii Colorectal Cancer Studies 2 and 3.; DACHS: Darmkrebs: Chancen der Verhütung durch Screening; DALS: Diet, Activity and Lifestyle Study; HPFS:Health Professionals Follow-up Study; HPFS_AD: Health Professionals Follow-up Study for colorectal adenoma; MEC: Multiethnic Cohort Study; NHS: Nurses’ Health Study; NHS_AD: Nurses’ Health Study for colorectal adenoma ; OFCCR: Ontario Familial Colorectal Cancer Registry; PMH-CCFR: Postmenopausal Hormone study- Colon Cancer Family Registry; PLCO: Prostate, Lung, Colorectal and Ovarian Cancer Screening Trial; VITAL: VITamins And Lifestyle; WHI: Women’s Health Initiative.

^b^: CCFR is a collaborating study with GECCO
