# Supplementary material for: Genome-Wide Interaction Analyses between Genetic Variants and Alcohol Consumption and Smoking for Risk of Colorectal Cancer
Source: PLoS Genet. 2016 Oct 10;12(10):e1006296. doi: 10.1371/journal.pgen.1006296 (PMC5065124; doi:10.1371/journal.pgen.1006296)
Supplement: S3 Table — (DOCX) [file pgen.1006296.s005.docx]

**S3 Table: Significant findings for genome-wide interaction analyses with alcohol consumption.**

|  |  |  |  |  |  | **SNP × light-to-moderate drinkers** | | |  | **SNP × heavy drinkers** | | |  | **LD R^2^ ^h^** | | **Impute r^2^** |
| --- | --- | --- | --- | --- | --- | --- | --- | --- | --- | --- | --- | --- | --- | --- | --- | --- |
| **SNP** | **Chr ^a^** | **BP ^b^** | **Gene** | **CA ^c^** | **MAF ^d^** | **OR (95% CI)^e^** | **P** | **P.Het ^f^** |  | **OR(95% CI)^e^** | **P** | **P.Het^f^** | **P^g^** |  |  |  |
| rs9409565 | 9 | 97251034 | *HIATL1* | C | 0.34 | 1.35 (1.21-1.49) | 1.76E-08 | 0.804 |  | 1.23 (1.04-1.45) | 0.017 | 0.283 | 1.23E-07 | | - | 0.98 |
| rs9409567 | 9 | 97252505 | *HIATL1* | A | 0.34 | 0.74 (0.67-0.82) | 1.78E-08 | 0.813 |  | 0.81 (0.69-0.96) | 0.016 | 0.282 | 1.24E-07 | | 1.0 | 0.99 |
| rs11789704 | 9 | 97233408 | *HIATL1* | C | 0.31 | 1.35 (1.21-1.5) | 2.19E-08 | 0.618 |  | 1.16 (0.97-1.37) | 0.096 | 0.337 | 1.40E-07 | | 0.9 | 1.00 |
| rs9409550 | 9 | 97223294 | *HIATL1* | C | 0.31 | 1.35 (1.21-1.5) | 2.43E-08 | 0.586 |  | 1.16 (0.97-1.37) | 0.098 | 0.346 | 1.55E-07 | | 0.9 | 1.00 |
| rs2139189 | 9 | 97238538 | *HIATL1* | A | 0.31 | 1.35 (1.21-1.5) | 2.62E-08 | 0.605 |  | 1.15 (0.97-1.37) | 0.100 | 0.362 | 1.66E-07 | | 0.9 | 1.00 |
| rs4744345 | 9 | 97207669 | *HIATL1* | A | 0.31 | 0.74 (0.67-0.82) | 2.69E-08 | 0.576 |  | 0.86 (0.73-1.03) | 0.094 | 0.322 | 1.73E-07 | | 0.9 | 0.99 |
| rs9409546 | 9 | 97209066 | *HIATL1* | C | 0.31 | 0.74 (0.67-0.82) | 2.70E-08 | 0.575 |  | 0.86 (0.73-1.03) | 0.097 | 0.328 | 1.72E-07 | | 0.9 | 0.99 |
| rs9409778 | 9 | 97242839 | *HIATL1* | A | 0.31 | 0.74 (0.67-0.82) | 2.84E-08 | 0.596 |  | 0.87 (0.73-1.03) | 0.100 | 0.366 | 1.80E-07 | | 0.9 | 1.00 |
| rs639276 | 9 | 97206737 | *HIATL1* | G | 0.31 | 1.35 (1.21-1.5) | 2.96E-08 | 0.573 |  | 1.16 (0.97-1.37) | 0.098 | 0.324 | 1.89E-07 | | 0.9 | 0.99 |
| rs9409564 | 9 | 97248166 | *HIATL1* | C | 0.31 | 1.34 (1.21-1.49) | 3.63E-08 | 0.623 |  | 1.16 (0.97-1.37) | 0.098 | 0.358 | 2.32E-07 | | 0.9 | 0.99 |
| rs1752784 | 9 | 97177826 | *HIATL1* | C | 0.31 | 1.34 (1.21-1.49) | 0.296 | 0.55 |  | 1.15 (0.97-1.37) | 0.106 | 0.240 | 2.57E-07 | | 0.8 | 0.98 |

^a^: Chr, chromosome; ^b^: BP, base pair position based on NCBI Build37; ^c^: CA, coded allele; ^d^: MAF, minor allele frequency; ^e^: Odds ratio and 95% confidence interval of the interaction term in models ^f^: P.Het, p value for heterogeneity test; ^g^ : the interaction p-value for light/moderate and heavy combined; ^h^:LD R^2^ with rs9409565.
