# Supplementary material for: Genome-Wide Interaction Analyses between Genetic Variants and Alcohol Consumption and Smoking for Risk of Colorectal Cancer
Source: PLoS Genet. 2016 Oct 10;12(10):e1006296. doi: 10.1371/journal.pgen.1006296 (PMC5065124; doi:10.1371/journal.pgen.1006296)
Supplement: S4 Table — (DOCX) [file pgen.1006296.s006.docx]

**S4 Table: Stratification analyses^a^ by alcohol consumption for the association of CRC with rs9409565 in men and women and by cancer rite.**

|  | **Men** | | **Women** | | **Colon** | | **Rectum^c^** | |
| --- | --- | --- | --- | --- | --- | --- | --- | --- |
| **Alcohol consumption** | **OR (95% CI)** | **P value** | **OR (95% CI)** | **P value** | **OR (95% CI)** | **P value** | **OR (95% CI)** | **P value** |
| Non/occasional | 0.87 (0.75-1.00) | 4.4E-02 | 0.86 (0.79-0.94) | 1.1E-03 | 0.86 (0.79-0.94) | 0.0006 | 0.83 (0.71-0.97) | 0.0177 |
| Light-to-moderate | 1.19 (1.09-1.31) | 2.7E-04 | 1.13 (1.02-1.24) | 1.5E-02 | 1.14 (1.06-1.24) | 0.0009 | 1.23 (1.09-1.40) | 0.0011 |
| Heavy | 1.15 (0.97-1.37) | 0.11 | 0.85 (0.61-1.19) | 0.34 | 1.07 (0.90-1.28) | 0.4498 | 1.03 (0.80-1.32) | 0.8298 |
| P-interaction^b^ | Light-to-moderate | 6.88E-05 |  | 3.73E-05 |  | 2.71E-06 |  | 0.00013 |
|  | Heavy | 0.0089 |  | 0.696 |  | 0.055 |  | 0.29 |
|  | Overall | 0.00029 |  | 0.00018 |  | 1.61E-05 |  | 0.00046 |

^a^: additive genetic models were used in analyses; ^b^: P value of interaction term between SNP and alcohol consumption.

^c^: Study with <25 rectal cases was excluded.
