# Supplementary material for: Genome-Wide Interaction Analyses between Genetic Variants and Alcohol Consumption and Smoking for Risk of Colorectal Cancer
Source: PLoS Genet. 2016 Oct 10;12(10):e1006296. doi: 10.1371/journal.pgen.1006296 (PMC5065124; doi:10.1371/journal.pgen.1006296)
Supplement: S5 Table — (DOCX) [file pgen.1006296.s007.docx]

**S5 Table: Interaction between rs9409565 and alcohol consumption for CRC risk based on one reference group and stratified by genotype (last two rows) and by alcohol consumption (last column).**

| **Alcohol consumption** |  | | **rs9409565 genotype** | | | | | **OR(95% CI) per C allele within strata of alcohol consumption** |
| --- | --- | --- | --- | --- | --- | --- | --- | --- |
|  | **TT** | | | **CT** | | **CC** | |  |
|  | **N Ca/Co** | **OR(95% CI)** | | **N Ca/Co** | **OR(95% CI)** | **N Ca/Co** | **OR(95% CI)** |  |
| **Non/occasional drinker** | 425/387 | 1 ^a^ | | 1495/1575 | 0.85 (0.73-1.00) | 1365/1593 | 0.75 (0.64-0.88) | 0.86 (0.80-0.93) |
|  |  |  | |  | P= 0.057 |  | P= 0.00042 | P= 0.00012 |
| **Light-to-moderate drinker** | 434/590 | 0.62 (0.51-0.75) | | 1646/2002 | 0.70 (0.60-0.83) | 1638/1717 | 0.83 (0.70-0.97) | 1.16 (1.08-1.24) |
|  |  | P= 1.3e-06 | |  | P= 1.7e-05 |  | P= 0.02 | P= 1.6e-05 |
|  |  |  | |  |  |  |  |  |
|  |  |  | |  |  |  |  |  |
| **ORs (95% CI) for light-to-moderate vs. non/occasional drinker within each genotype** | 859/977 | 0.62 (0.51-0.75) | | 3141/3577 | 0.82 (0.74-0.91) | 3003/3310 | 1.11 (1.00-1.23) | - |
|  |  | P= 1.3e-06 | |  | P= 0.00021 |  | P= 0.059 | - |

^a^: non/occasional drinkers with rs9409565 TT genotype as the reference group. Non-/occasional drinkers: drinking < 1 gram of alcohol per day; light-to-moderate drinkers: drinking 1-28 grams of alcohol per day; and heavy drinkers: drinking >28 grams of alcohol per day. Men and women were analyzed separately in each study and age, study site (if applicable), and population structure were adjusted in model. Note: N Ca/Co, number of case/control.
