# Supplementary material for: Genome-Wide Interaction Analyses between Genetic Variants and Alcohol Consumption and Smoking for Risk of Colorectal Cancer
Source: PLoS Genet. 2016 Oct 10;12(10):e1006296. doi: 10.1371/journal.pgen.1006296 (PMC5065124; doi:10.1371/journal.pgen.1006296)
Supplement: S6 Table — (DOCX) [file pgen.1006296.s008.docx]

**S6 Table: Interactions between rs9409565 and alcohol consumption for CRC risk using Empirical Bayesian (EB) interaction analysis, case-control (CC) logistic regression and case-only (CO) interaction analysis.**

| SNP | Type^a^ | OR^b^ | p-value^c^ |  |  |  |  |  |
| --- | --- | --- | --- | --- | --- | --- | --- | --- |
| rs9409565 | EB | 1.25 (1.14-1.37) | 2.67E-06 |  |  |  |  |  |
| rs9409565 | CC | 1.35 (1.22-1.50) | 1.42E-08 |  |  |  |  |  |
| rs9409565 | CO | 1.11 (1.03-1.20) | 0.007807 |  |  |  |  |  |

^a^EB: Empirical Bayesian interaction analysis; CC: Case-control logistic regression; CO: Case-only interaction analysis. All analyses were done by men and women separately in each study and age, study site (if applicable), and population structure were adjusted in models.

^b^Non/occasional drinkers as the reference group. The ORs represent the ORs of the interactions between SNP and light-to-moderate drinkers compare to reference group.

^c^The p value of the interaction between SNP and alcohol consumption.
