# Supplementary material for: Genome-Wide Interaction Analyses between Genetic Variants and Alcohol Consumption and Smoking for Risk of Colorectal Cancer
Source: PLoS Genet. 2016 Oct 10;12(10):e1006296. doi: 10.1371/journal.pgen.1006296 (PMC5065124; doi:10.1371/journal.pgen.1006296)
Supplement: S7 Table — (DOCX) [file pgen.1006296.s009.docx]

**S7 Table: The association between CRC and alcohol consumption stratified by cancer site.**

| **Site** | **Description** | **OR** | **95% C.I.** | **p** | **p.het** |
| --- | --- | --- | --- | --- | --- |
| Colon | heavy vs. non/occasional drinker | 1.17 | (1.04, 1.32) | 8.03E-03 | 0.42 |
| Colon | light-to-moderate vs. non/occasional drinker | 0.90 | (0.83, 0.96) | 2.59E-03 | 0.93 |
| Rectum | heavy vs. non/occasional drinker | 1.35 | (1.17, 1.55) | 2.66E-05 | 0.12 |
| Rectum | light-to-moderate vs. non/occasional drinker | 0.89 | (0.82, 0.97) | 5.13E-03 | 0.18 |

Note: Non-/occasional drinkers: drinking < 1 gram of alcohol per day; light-to-moderate drinkers: drinking 1-28 grams of alcohol per day; and heavy drinkers: drinking >28 grams of alcohol per day. OR: odds ratio; C.I.: confidential interval; p: p value of meta-analysis; p.het: p value of heterogeneity.
