# Supplementary material for: Genome-Wide Interaction Analyses between Genetic Variants and Alcohol Consumption and Smoking for Risk of Colorectal Cancer
Source: PLoS Genet. 2016 Oct 10;12(10):e1006296. doi: 10.1371/journal.pgen.1006296 (PMC5065124; doi:10.1371/journal.pgen.1006296)
Supplement: S8 Table — (DOCX) [file pgen.1006296.s010.docx]

**S8 Table: Expression Quantitative Trait Locus tagged by rs9409565 for genes in 1Mb.**

| **PMID** | **Tissue** | **Correlated gene** | **p-value** |
| --- | --- | --- | --- |
| 25954001 | Cells_Transformed_fibroblasts | HIATL1 | 9.14E-12 |
| 25954001 | Esophagus_Muscularis | HIATL1 | 1.01E-07 |
| 25954001 | Skin_Sun_Exposed_Lower_leg | HIATL1 | 2.89E-07 |
| 25954001 | Spleen | RP11-307E17.8 | 2.79E-06 |
| 25954001 | Whole_Blood | HIATL1 | 1.54E-12 |
| 24037378 | Lymphoblastoid_EUR_exonlevel | ENSG00000148110.10_97177440_97177535 | 1.84E-06 |
| 24037378 | Lymphoblastoid_EUR_exonlevel | ENSG00000148110.10_97191471_97191533 | 4.02E-07 |
| 24037378 | Lymphoblastoid_EUR_exonlevel | ENSG00000148110.10_97200683_97200810 | 2.58E-12 |
| 24037378 | Lymphoblastoid_EUR_exonlevel | ENSG00000148110.10_97203267_97203381 | 2.58E-16 |
| 24037378 | Lymphoblastoid_EUR_exonlevel | ENSG00000148110.10_97207246_97207464 | 2.67E-12 |
| 24037378 | Lymphoblastoid_EUR_exonlevel | ENSG00000148110.10_97209105_97209218 | 8.14E-13 |
| 24037378 | Lymphoblastoid_EUR_exonlevel | ENSG00000148110.10_97213738_97213809 | 2.58E-11 |
| 24037378 | Lymphoblastoid_EUR_exonlevel | ENSG00000148110.10_97216240_97216352 | 1.58E-10 |
| 24037378 | Lymphoblastoid_EUR_exonlevel | ENSG00000148110.10_97218522_97218619 | 1.91E-11 |
| 24037378 | Lymphoblastoid_EUR_exonlevel | ENSG00000148110.10_97220604_97220746 | 4.85E-09 |
| 24037378 | Lymphoblastoid_EUR_exonlevel | ENSG00000148110.10_97221442_97223324 | 1.59E-21 |
| 24037378 | Lymphoblastoid_EUR_genelevel | HIATL1 | 1.26E-08 |
| 24013639 | Whole_Blood | HIATL1 | 7.00E-138 |
| 24013639 | Whole_Blood | ZNF169 | 4.25E-07 |
| … | Colon adenocarcinoma (COAD) | C9orf130 | 0.005 |
| … | Colon adenocarcinoma (COAD) | FGD3 | 0.04 |
