# Supplementary material for: Genome-Wide Interaction Analyses between Genetic Variants and Alcohol Consumption and Smoking for Risk of Colorectal Cancer
Source: PLoS Genet. 2016 Oct 10;12(10):e1006296. doi: 10.1371/journal.pgen.1006296 (PMC5065124; doi:10.1371/journal.pgen.1006296)
Supplement: S1 Fig — (DOCX) [file pgen.1006296.s011.docx]

**S1 Fig: Association between CRC risk and light/moderate drinker vs non/occasional drinker, stratified by genotype across studies** (the interaction estimates and p-values are slightly different from those shown in Table 1 because the Forest plots are based on three separate stratified analyses while results in Table 1 are derived from a single joint effect analysis)
