# Supplementary material for: Genome-Wide Interaction Analyses between Genetic Variants and Alcohol Consumption and Smoking for Risk of Colorectal Cancer
Source: PLoS Genet. 2016 Oct 10;12(10):e1006296. doi: 10.1371/journal.pgen.1006296 (PMC5065124; doi:10.1371/journal.pgen.1006296)
Supplement: S2 Fig — The 4 probes for HIATL1 all showed that HIATL1 expression was significantly higher in tumor tissue than in normal tissue (Paired t test, P = 4.4×10^−9^ to 7.2×10^−5^); the results from two probes that uniquely match HIATL1 transcript were shown in a (P = 7.2×10^−5^) & b (P = 5.1×10^−7^); These results were replicated in the colorectal tumor-normal-matched samples from TCGA (c,d) (P = 0.025). In figures a, b, and c each line represent a colorectal cancer case connecting the values of gene expression in adjacent normal tissue to tumor tissue from that same case. In figure d the log2 transformed mean expression with 95% confidence interval is shown with a line connecting values of gene expression in tumor and adjacent normal tissue. (DOCX) [file pgen.1006296.s012.docx]

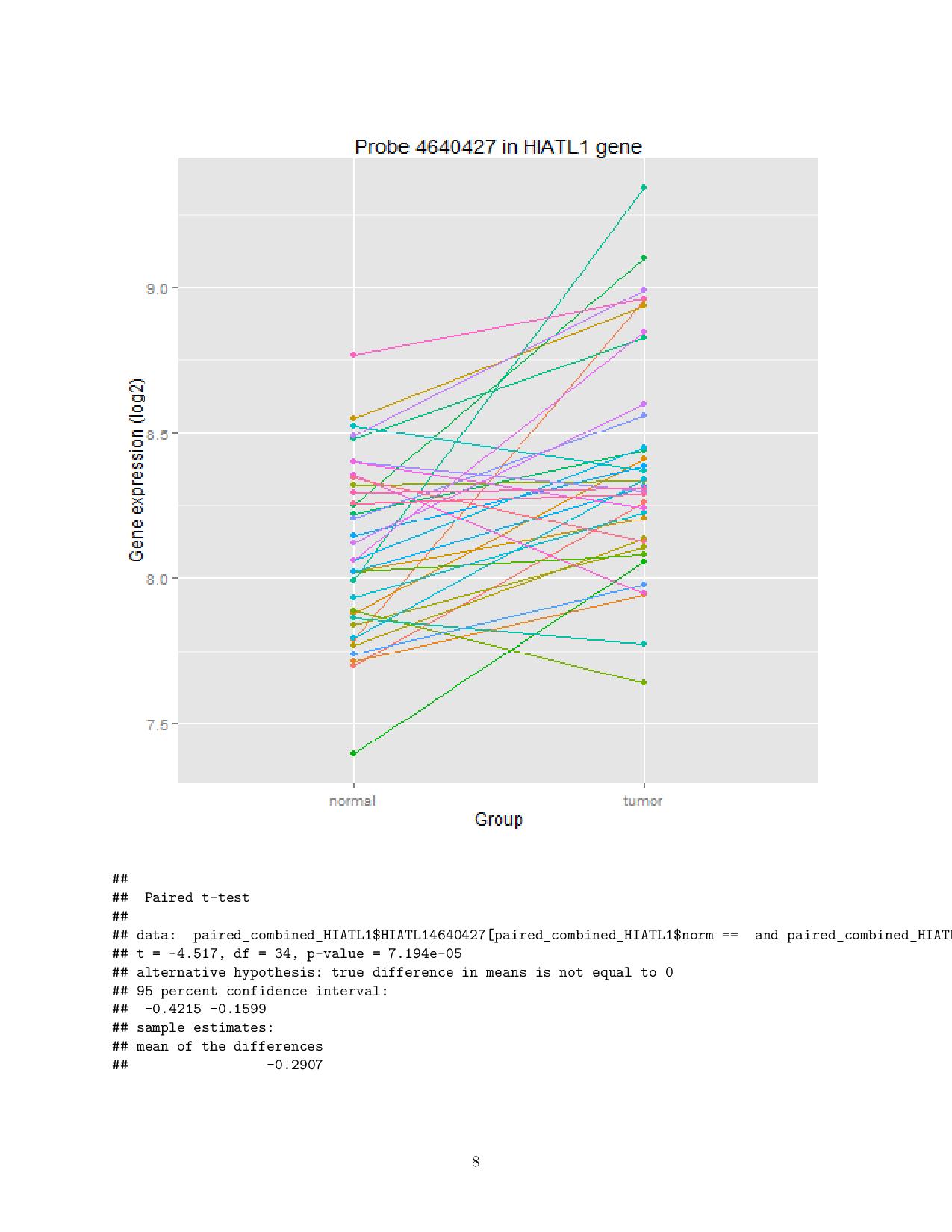

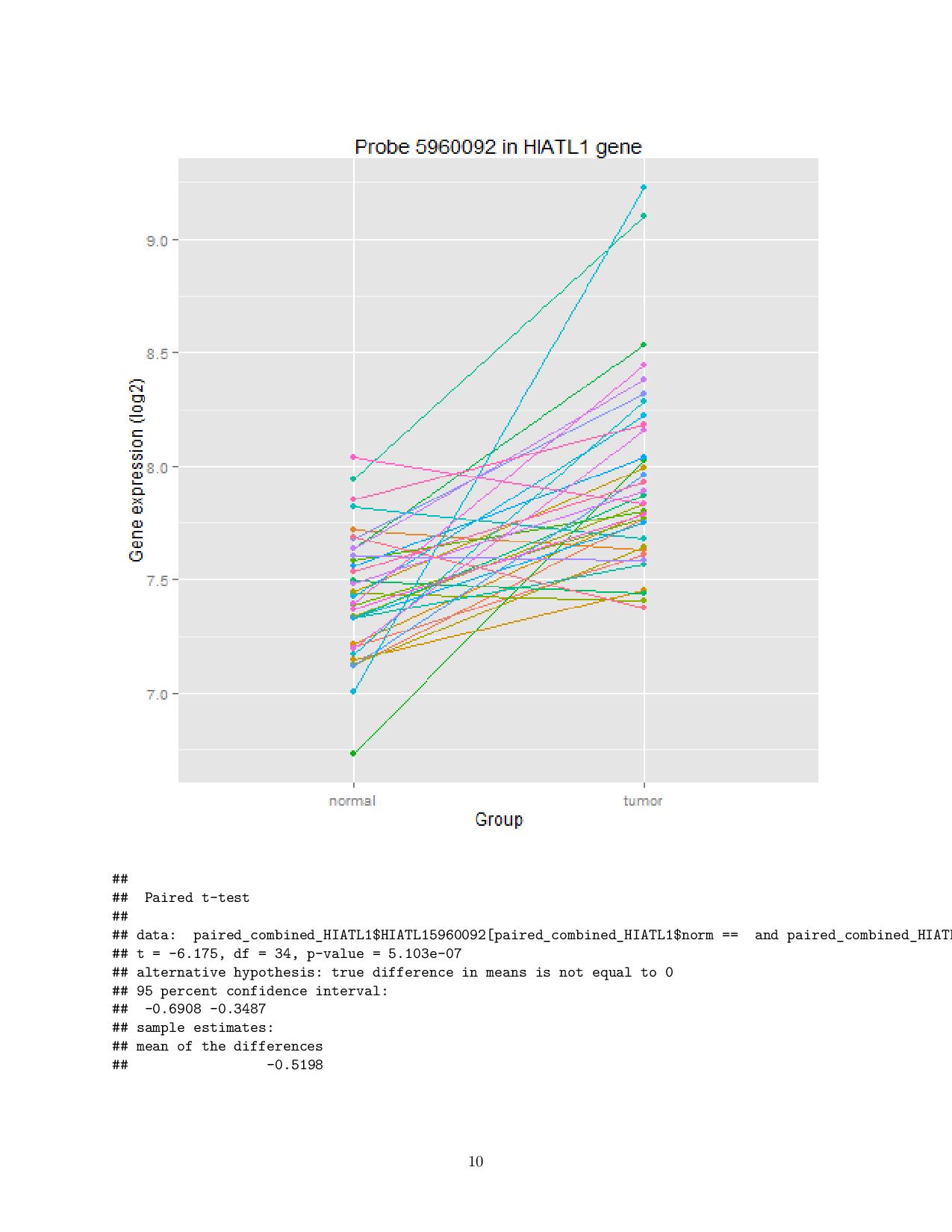


**(a) (b)**


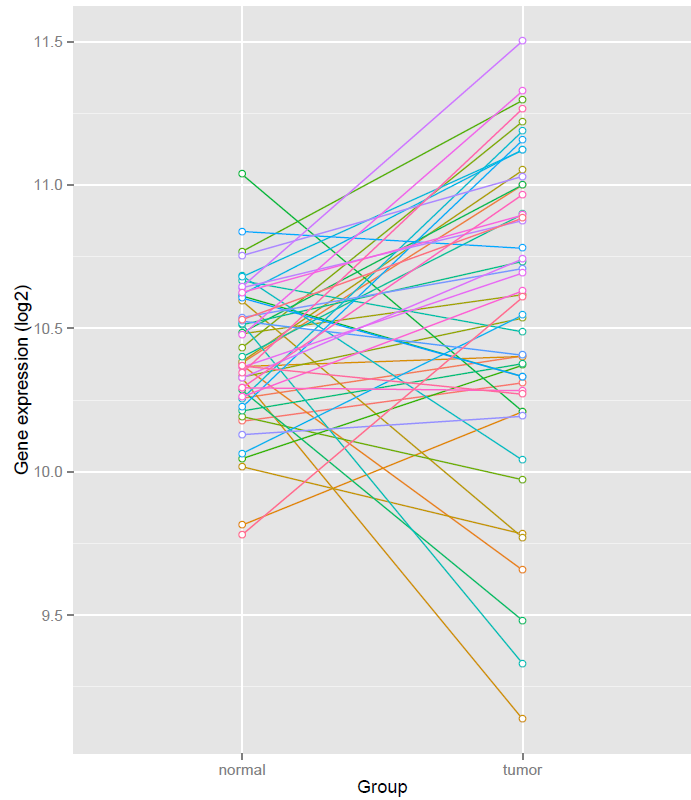

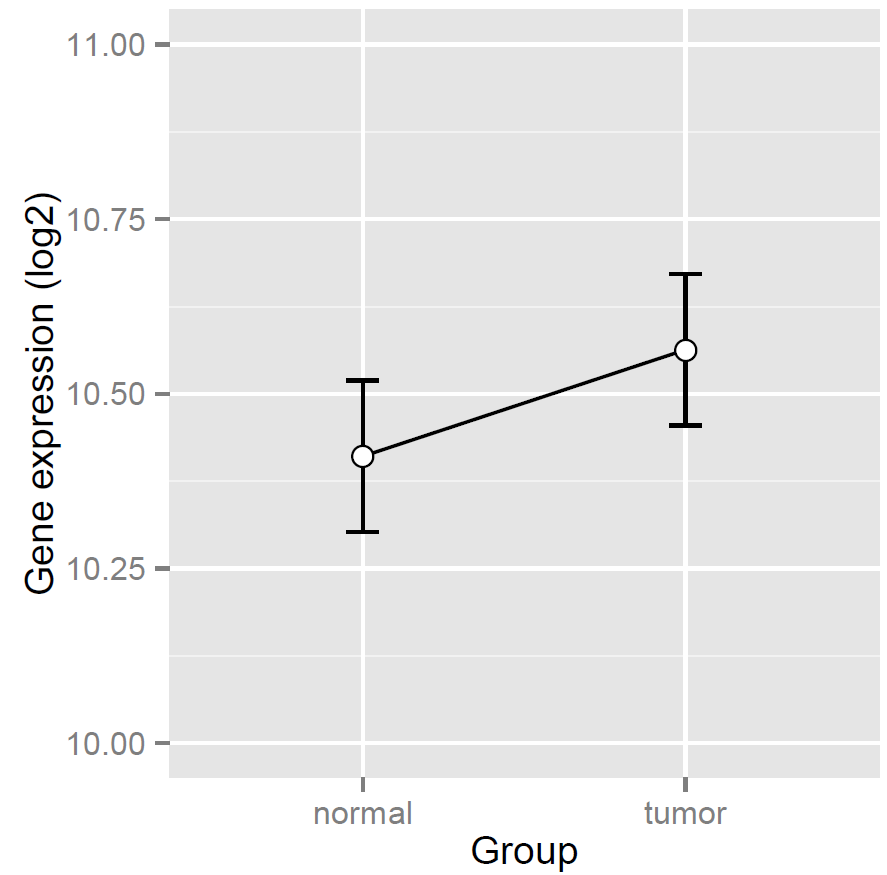


**(c) (d)**

**S2 Fig: Gene expression levels of *HIATL1* in colorectal tumor tissue and paired adjacent normal tissue from 35 colorectal cancer cases in ColoCare (a,b) and 50 colorectal cancer cases in TCGA (c,d).** The 4 probes for *HIATL1* all showed that *HIATL1* expression was significantly higher in tumor tissue than in normal tissue (Paired t test, P=4.4×10^-9^ to 7.2×10^-5^); the results from two probes that uniquely match *HIATL1* transcript were shown in a (P=7.2×10^-5^) & b (P=5.1×10^-7^); These results were replicated in the colorectal tumor-normal-matched samples from TCGA (c,d) (P=0.025). In figures a, b, and c each line represent a colorectal cancer case connecting the values of gene expression in adjacent normal tissue to tumor tissue from that same case. In figure d the log2 transformed mean expression with 95% confidence interval is shown with a line connecting values of gene expression in tumor and adjacent normal tissue.
