# Supplementary material for: Genome-Wide Interaction Analyses between Genetic Variants and Alcohol Consumption and Smoking for Risk of Colorectal Cancer
Source: PLoS Genet. 2016 Oct 10;12(10):e1006296. doi: 10.1371/journal.pgen.1006296 (PMC5065124; doi:10.1371/journal.pgen.1006296)
Supplement: S4 Fig — The alcohol consumption at reference time was categorized into four groups ([0, 4.7), [4.7, 12.5), [12.5, 25.3), & > = 25.3 grams of alcohol/day). Y axis: normalized and log2 transformed values of gene expression; X axis: the lifetime alcohol consumption (grams of alcohol/day). Each dot in the figure represented a single sample. (a) and (b) represented the results from two probes uniquely matching HIATL1 transcript. (DOCX) [file pgen.1006296.s014.docx]

**
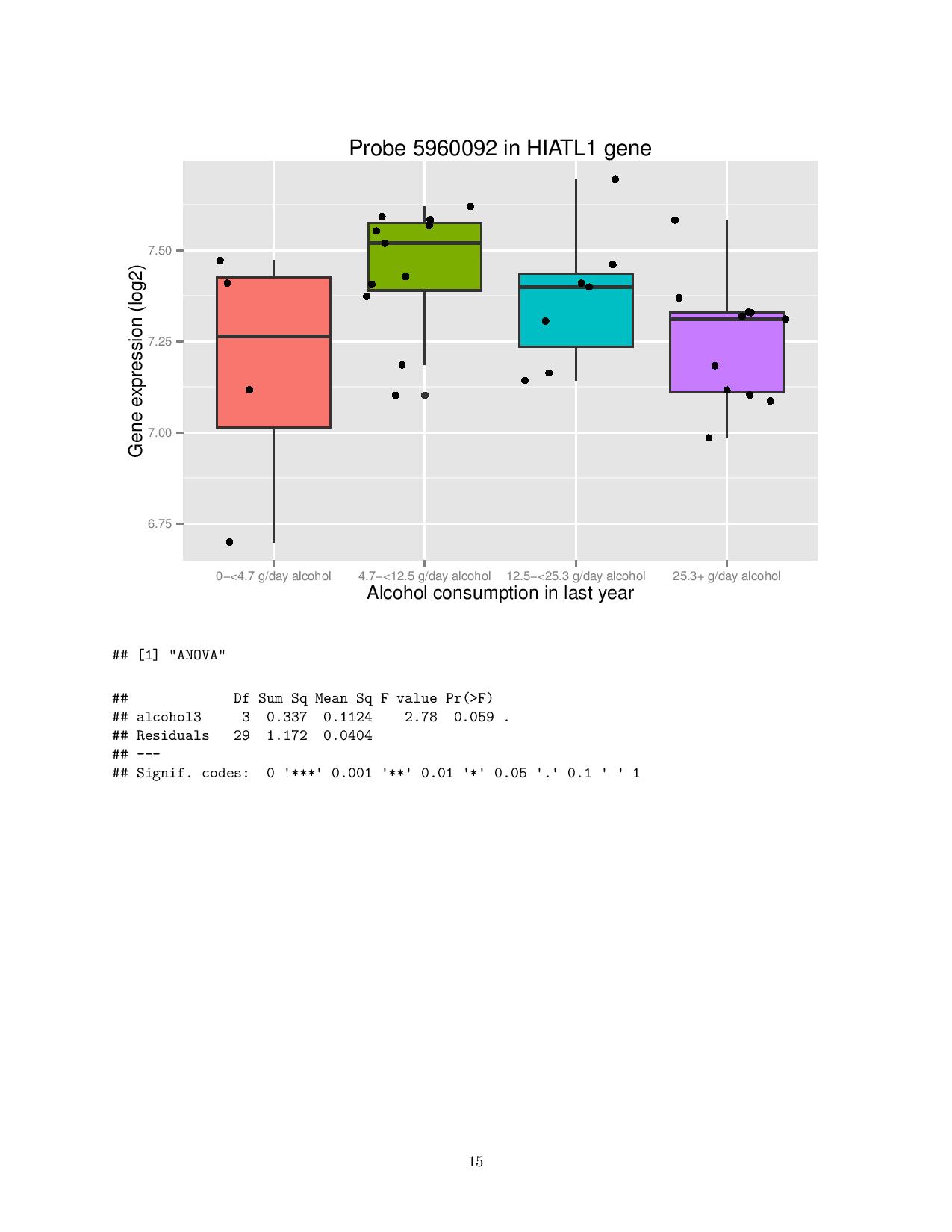

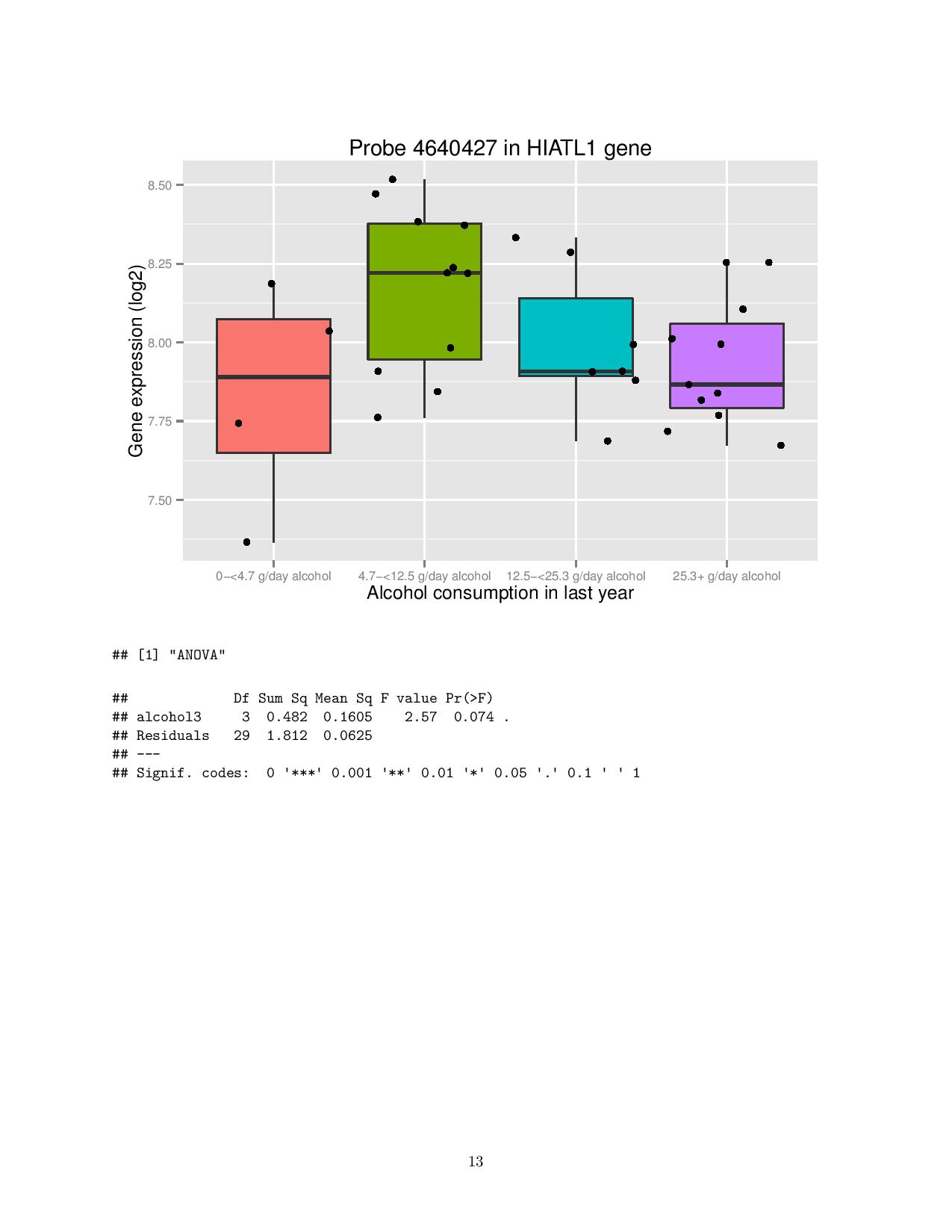
**

(a) (b)

**S4 Fig: The analysis of variance (ANOVA) to test differences in the expression of *HIATL1* between different levels of alcohol consumption at reference time (the year before enrollment) in 33 normal colon tissues (a: P value=0.06; b: P value=0.07).** The alcohol consumption at reference time was categorized into four groups ([0, 4.7), [4.7, 12.5), [12.5, 25.3), & >=25.3 grams of alcohol/day). Y axis: normalized and log2 transformed values of gene expression; X axis: the lifetime alcohol consumption (grams of alcohol/day). Each dot in the figure represented a single sample. (a) and (b) represented the results from two probes uniquely matching *HIATL1* transcript.
