# Supplementary material for: Genome-Wide Interaction Analyses between Genetic Variants and Alcohol Consumption and Smoking for Risk of Colorectal Cancer
Source: PLoS Genet. 2016 Oct 10;12(10):e1006296. doi: 10.1371/journal.pgen.1006296 (PMC5065124; doi:10.1371/journal.pgen.1006296)
Supplement: S5 Fig — Individual genotypes are plotted on a strip chart, where observed and permuted P values are labeled. r: Spearman's rho; P: observed P value; Pemp: p value of 10,000 permutations. (DOCX) [file pgen.1006296.s015.docx]

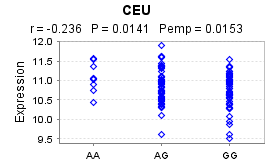


**S5 Fig: The associations between rs9409567 and *HIATL1* in eQTL (expression quantitative trait loci) study (Stranger BE, et al. (2012) Patterns of cis regulatory variation in diverse human populations. PLoS Genetics.) among the Utah residents with Northern and Western European ancestry (CEU, n=109) from Genevar (GENe Expression VARiation) in the Wellcome Trust Sanger Institute.** Individual genotypes are plotted on a strip chart, where observed and permuted P values are labeled. r: Spearman's rho; P: observed P value; Pemp: p value of 10,000 permutations.
