# Supplementary material for: Genome-Wide Interaction Analyses between Genetic Variants and Alcohol Consumption and Smoking for Risk of Colorectal Cancer
Source: PLoS Genet. 2016 Oct 10;12(10):e1006296. doi: 10.1371/journal.pgen.1006296 (PMC5065124; doi:10.1371/journal.pgen.1006296)
Supplement: S6 Fig — rs9409565 (shown as green bar) is correlated with 142 variants (r2≥0.5 in 1000 Genomes Phase 3 European populations). The tagged variants span across intronic regions and approximately 50kb downstream and 75kb upstream of HIATL1. Eigtheen of these variants fall within a transcriptionally active region in colorectal tissue, and four of these variants (rs7042481,rs7858085, rs9409510, rs28406858) are positioned within three variant enhancer loci (VEL, shown as orange bars). (DOCX) [file pgen.1006296.s016.docx]

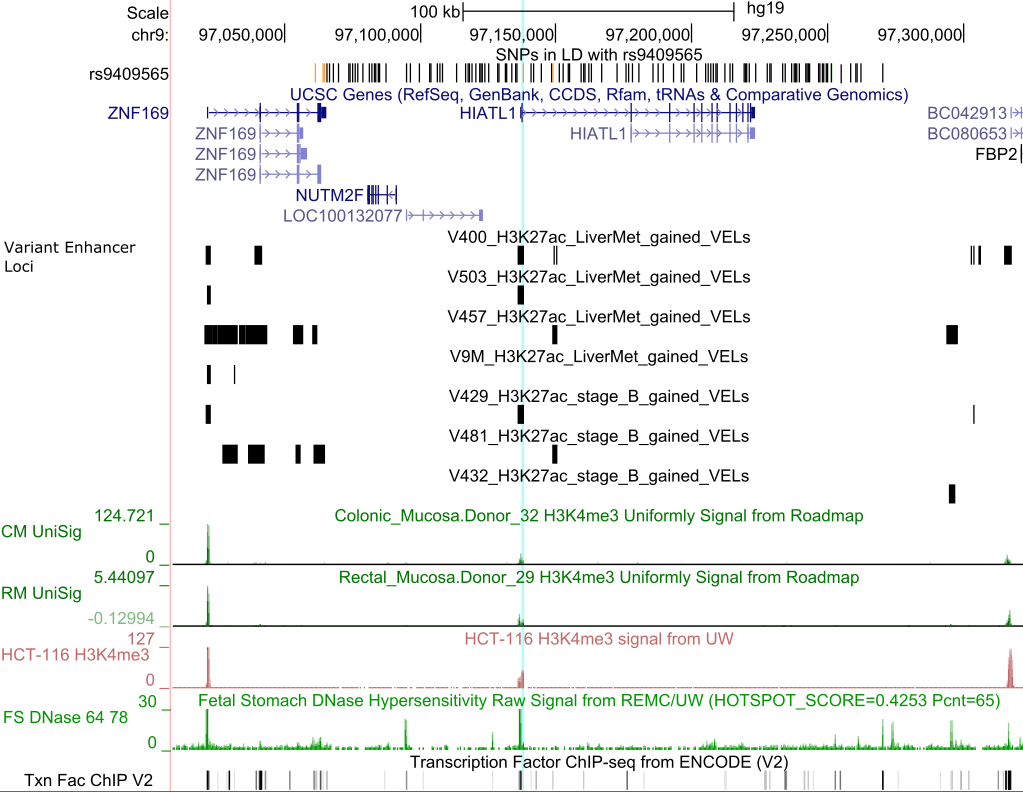


**S6 Fig: Functional annotation of rs9409567 and correlated SNPs in chromosome 9.** rs9409565 (shown as green bar) is correlated with 142 variants (r^2^≥0.5 in 1000 Genomes Phase 3 European populations). The tagged variants span across intronic regions and approximately 50kb downstream and 75kb upstream of *HIATL1*. Eigtheen of these variants fall within a transcriptionally active region in colorectal tissue, and four of these variants (rs7042481,rs7858085, rs9409510, rs28406858) are positioned within three variant enhancer loci (VEL, shown as orange bars).
