# Supplementary material for: Genome-Wide Interaction Analyses between Genetic Variants and Alcohol Consumption and Smoking for Risk of Colorectal Cancer
Source: PLoS Genet. 2016 Oct 10;12(10):e1006296. doi: 10.1371/journal.pgen.1006296 (PMC5065124; doi:10.1371/journal.pgen.1006296)
Supplement: S7 Fig — Rs28406858 is shown as the orange bar and highlighted in blue. The variant is positioned in both a variant enhancer locus in the first intron of HIATL1 and a protein binding site for ELF1. Bioinformatic annotation suggests this variant is a strong candidate for functional follow-up. (DOCX) [file pgen.1006296.s017.docx]

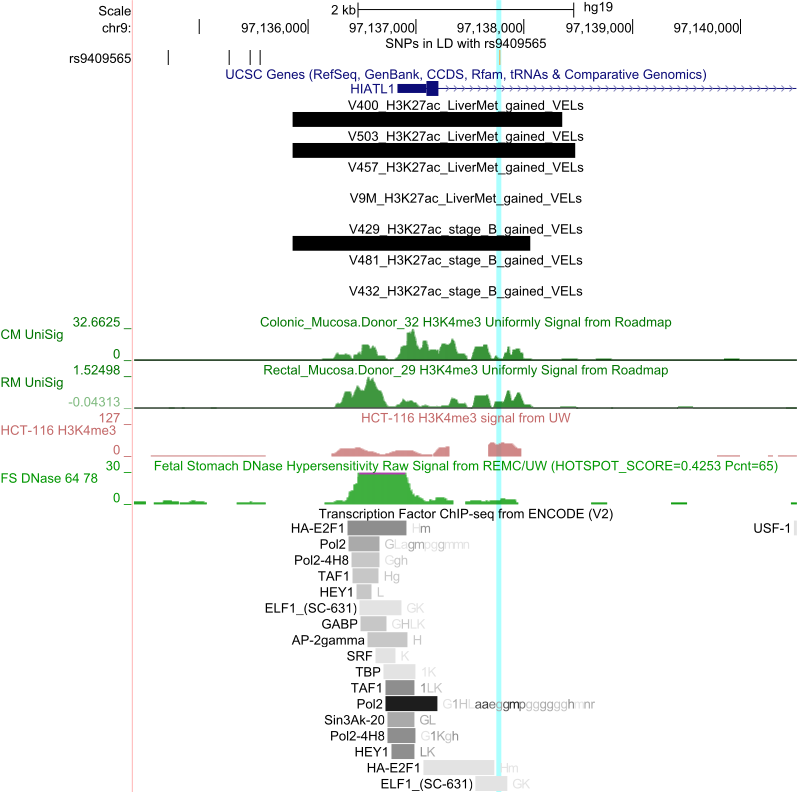


**S7 Fig**: **Functional annotation of rs28406858 in the rs9409567 locus.** Rs28406858 is shown as the orange bar and highlighted in blue. The variant is positioned in both a variant enhancer locus in the first intron of *HIATL1* and a protein binding site for ELF1. Bioinformatic annotation suggests this variant is a strong candidate for functional follow-up.
